# Subcritical Water and Pressurised Ethanol Extractions for Maximum Recovery of Antioxidants from Orange Peel Herbal Dust with Evaluation of Its Pharmacological Potential Using In Silico and In Vitro Analysis

**DOI:** 10.3390/antiox14060638

**Published:** 2025-05-26

**Authors:** Slađana Krivošija, Ana Ballesteros-Gómez, Mire Zloh, Nataša Milić, Aleksandra Popović, Nataša Nastić, Senka Vidović

**Affiliations:** 1 Department of Pharmaceutical Engineering, Faculty of Technology Novi Sad, University of Novi Sad, Boulevard Cara Lazara 1, 21000 Novi Sad, Serbia; sladjana.krivosija@uns.ac.rs (S.K.); natasa.nastic@uns.ac.rs (N.N.); 2 Department of Analytical Chemistry, Institute of Chemistry for Energy and the Environment, University of Córdoba, 14071 Cordoba, Spain; ana.ballesteros@uco.es; 3 UCL School of Pharmacy, University College London, 29/39 Brunswick Square, London WC1N 1AX, UK; mire.zloh@ffns.ac.rs; 4 Department of Pharmacy, Faculty of Medicine, University of Novi Sad, Hajduk Veljkova 3, 21000 Novi Sad, Serbia; natasa.milic@mf.uns.ac.rs; 5 Department of Physiology, Faculty of Medicine, University of Novi Sad, Hajduk Veljkova 3, 21000 Novi Sad, Serbia; aleksandra.rakovac@mf.uns.ac.rs

**Keywords:** pressurised liquid extraction, subcritical water extraction, pressurised ethanol extraction, orange peel herbal dust, waste valorisation, antitumor activity

## Abstract

This research explored the potential of pressurised liquid extraction techniques for valorising herbal orange peel dust (OPD) waste from the filter tea industry. A series of experiments were conducted, varying the temperature (120–220 °C) and solvent (water and 50% (*v*/*v*) ethanol), while pressure and time were kept constant. Afterward, the obtained extracts were analysed by LC-ESI-MS/MS for determining the chemical composition. The highest concentrations of the most dominant compounds, the antioxidants hesperidin (662.82 ± 22.11 mg/L) and naringin (62.37 ± 2.05 mg/L), were found at specific temperatures using subcritical water extraction. In silico studies indicated that these compounds could interact with sirtuin-1 and growth factor beta receptors, suggesting potential anti-ageing benefits for skin. In vitro experiments on rat hepatoma cells (H4IIE) revealed that OPD extracts had antitumor potential, inhibiting cell proliferation and altering cell morphology. These findings underscore the importance of temperature and extraction technique in obtaining antioxidant-rich extracts with pharmacological potential. The resulting extracts, obtained using green solvents, show promise for cosmetic applications, though further in vivo studies are needed to confirm their therapeutic efficacy.

## 1. Introduction

For decades, traditional herbal medicine has played a vital role in the treatment of numerous diseases. High-value bioactive compounds found in herbs represent the basis for the prevention and treatment of various diseases [1]. The thousands of years old extraction process has been improved to such an extent that not only has the maximum use of bioactive principles from plant material been achieved, but on the other hand, the emphasis is now placed on the ecology, economic profitability, and sustainability of the processes themselves. The application of such technologies not only reduces or eliminates the use of organic solvents but also contributes to the safety, quality, and applicability of plant extracts [2].

Among the various new green extraction technologies that have been developed recently, pressurised liquid extraction (PLE) enjoys great scientific interest. This extraction technique is based on the use of solvents under pressurised conditions and at temperatures between their boiling and critical points. Various liquids are applicable in the PLE technique. Among them, subcritical water extraction (SWE) is considered the most promising [3]. This attractive technique involves the use of water in a subcritical state, which is achieved at temperatures between 100 and 374.15 °C and pressures high enough to keep water in a liquid state [4]. Water, in this case, replaces conventional organic, easily volatile solvents, and also represents an easily available, cheap, safe, and effective extractant. In SWE, the pressure does not have such a significant effect on the extraction process efficiency, as long as it is high enough to keep the water in a liquid state [3]. On the other hand, temperature is the most significant influencing factor, because with increasing temperature, there is better and easier mass transfer between medium and matrix, a higher solubility of dissolved substances, and a diffusion speed at lower solvent viscosity and surface tension [5,6]. Namely, with an increase in temperature, the physical and chemical properties of water can vary significantly, especially its dielectric constant [7,8]. Also, with the increase in this parameter, water changes from a polar to a non-polar solvent, which makes it a potential multifunctional solvent for extraction. What can also greatly affect the quality of SWE extracts is the extraction time. Long-term exposure to high temperatures can lead to the degradation of thermosensitive compounds or the creation of new antioxidants [6,9]. Consequently, in order to ensure the most efficient process possible, it is necessary to optimise the process conditions in terms of minimum energy consumption and operating costs, with maximum yield of bioactive compounds.

In PLE, it is also possible to use a mixture of water/ethanol and ethanol as solvents because they are also generally recognised as safe solvents [10]. Subcritical ethanol or ethanol under pressure is heated and maintained in a subcritical state under temperatures between 78.37 °C and 240.8 °C and pressures between 1 and 6.13 MPa [11]. The chemical properties of ethanol also change under the influence of temperature, as the viscosity, dielectric constant, and surface tension decrease with increasing temperature, while diffusivity increases. The effects of pressurised ethanol extraction (PEE) on product yield and quality may be different depending on operating conditions, and the obtained bioactive products represent a potentially important source of resources for the sectors of pharmaceuticals, food, etc. [12]. A study conducted by Mufari et al. [13] demonstrated the efficacy of subcritical extraction using an ethanol/water mixture to isolate the bioactive principles of germinated quinoa. Subcritical extraction using aqueous ethanol was also studied on defatted rice bran [14], and it improved the cold-water swelling ability of oat starch [15]. Moreover, subcritical treatment using ethanol and aqueous ethanol solution showed the ability to achieve a high total phenol content and antioxidant activity of rice stem, as well as *Arctium lappa* leaves [16,17]. However, scarce information has been reported on the extraction of bioactive compounds from orange peel using this type of extraction. Therefore, PEE, as another green technique, was chosen in order to investigate the influence of ethanol’s addition to SWE, as well as to compare both the yield and the profile of the total content of polyphenols from the herbal dust of orange peel.

Orange fruit (*Citrus sinensis* L.) is the most common citrus fruit grown worldwide, even being grown in over 130 countries. Orange peel waste obtained during the production of either juice or canned food is one of the most abundant wastes in the food industry. Annually, more than 76 thousand tons of oranges are produced worldwide [18], and consequently, due to large production or processing, a large amount of waste is created, even up to 50% of the total fruit weight [19], which further causes large economic–industrial costs. This valuable biowaste is underutilised and is mostly either dumped in landfills or used as compost [20,21].

However, considering the compositional profile of the peel, its transformation into value-added products is considered a promising option from an economic and ecological perspective [22]. Therefore, more and more work is being performed on the analysis of such raw material in an attempt to valorise it and reuse it. Orange peel is a chemically complex substrate and can be used for the extraction and isolation of high-value compounds like proteins, fibres, polysaccharides, phenolic compounds, aromatic compounds, vitamins, and carotenoids. Naringin and hesperidin stand out as the most abundant flavonoids in orange peel [23]. The bioactivity shown by the polyphenolic compounds of orange peel has long been known [24], and, in addition to the dominant antioxidant effect, may include chemopreventive, anti-inflammatory, and antimicrobial effects [25]. The citrus peel extracts have also shown considerable skin anti-ageing activity [26], with their components explored for the development of topical applications, including hesperidin as nanocrystals [27] and naringin in microemulsion formulations [28].

Orange peel is important in the production of herbal teas as an aromatic ingredient. In the process of filter tea production, orange peel dust is generated as food waste. The particle size of this powder is smaller than the pore size of the filter paper (<0.315 mm), and accordingly, it cannot be used for the production of filter tea and is considered waste. Although a certain loss of bioactivity of these materials has been observed, generally, herbal dusts still represent an exceptional source of bioactive compounds. This has already been confirmed in several studies conducted using different techniques for the valorisation of such waste [29,30,31].

The effectiveness of PLE for the extraction of bioactive compounds from orange peel herbal dust using a combination of pressurised water and ethanol has not, to our knowledge, been evaluated so far. Therefore, this study was designed to gain new insight into this topic and contribute to the development of new uses of herbal orange peel dust using more environmentally friendly techniques to reduce the amount of waste in the filter tea industry. Therefore, the effect of extraction temperature, as the most important parameter affecting the efficiency of SWEs and PEEs was evaluated in terms of its impact on the recovery, stability, and degradation of phenolic constituents. The obtained extracts were analysed using liquid chromatography–high resolution tandem mass spectrometry (LC-ESI-MS/MS) in order to identify and quantify the main polyphenolic compounds that also contribute to the use of extracts in one of the many branches of industry. Further, molecular docking was used as a tool that would highlight the obtained extract’s potential to affect the sirtuin-1 and growth factor beta (TGF-β) receptors, protein targets involved in pathways related to skin ageing. Finally, the antitumor potential of the extracts was evaluated using an in vitro model based on rat hepatoma cells (H4IIE) to further explore their pharmaceutical relevance.

## 2. Materials and Methods

### 2.1. Plant Material and Chemicals

The investigated material, orange peel herbal dust (*Citrus sinensis* L.), was obtained from the local filter tea factory Fructus d.o.o. (Bačka Palanka, Serbia). The orange peel dust (OPD) was created as waste during the process of cutting, grinding, and fractionating the raw material in the filter tea factory. The particle size of the material obtained in this way was <0.315 mm, while the moisture content in the obtained material was 3.36 ± 0.63%.

All reagents were of analytical grade. Gallic acid and Folin–Ciocalteu reagent were supplied by Sigma-Aldrich (Steinheim, Germany). Methanol was supplied by Riedel-de Haën (Seelze, Germany), while sodium carbonate was purchased from Aldrich (St. Louis, MO, USA). Ultrapure water was produced in an Elix^®^ Essential 3 water purification system from Merck Millipore (Madrid, Spain). Formic acid, ammonium formiate, and standards for phenolic compounds (hesperidin, naringin, p-coumaric acid, caffeic acid, and gallic acid) were acquired from Aldrich (St. Louis, MO, USA).

### 2.2. Pressurised Liquid Extraction

Both types of extraction—subcritical water extraction (SWE) and pressurised ethanol extraction (PEE)—were performed using a high-pressure serial extractor (Parr Instrument Company, Moline, IL, USA). The extractor was filled with 7 g of OPD plant material and 140 mL double-distilled water for SWE, while for PEE, 50% (*v*/*v*) ethanol was used as an extraction solvent. The independent variable for both types of extraction (SWE and PEE) was temperature (120–220 °C), while pressure (20 bar) and time (15 min) were kept constant. The obtained extracts were filtered through filter paper under a vacuum and stored at 4 °C until further analysis.

During further analyses, OPD extracts were named as SWE and PEE depending on the type of extraction by which they were obtained, as well as given numbers from 1 to 6 depending on the applied process temperature (from the lowest to the highest).

### 2.3. Determination of Extraction Yield and Total Phenol Content in the OPD Extracts

The extraction yield (EY) was determined by evaporating a known volume of solvent extract under vacuum. After that, drying was carried out at 105 °C until a constant mass was reached. All experiments were performed in triplicate and the results were expressed as mean values in percentage (%), i.e., the mass of the dry extract obtained (g) per 100 g of dry plant material.

The total phenol content (TPC) in OPD extracts, obtained by SWE or PEE, was determined by the Folin–Ciocalteu method. For TPC quantification, the SWE and PEE extracts were diluted 1:10 *v*/*v* with an adequate solvent (solvent used for extraction). After that, 50 µL of the diluted sample, 1.5 mL of distilled water, 100 µL of 0.1N Folin–Ciocalteu reagent, and 300 µL of sodium carbonate solution (200 g/L) were added to a 2 mL Eppendorf tube. After 90 min of incubation in the dark, absorbance was measured at 760 nm using a Thermo Spectronic Helios ε spectrophotometer from Lubbock (Madrid, Spain). Quantitative analysis was carried out using standard solutions of gallic acid prepared in ultrapure water in concentration range (5–1000 mg/L), which were then processed as reported before [32]. The results were expressed as gallic acid equivalents per gram of dry weight (mg GAE/g dw). All experiments were performed in triplicate and the results were expressed as mean values.

### 2.4. LC-ESI-MS/MS Analysis of OPD Extracts

OPD extracts obtained by SWE or PEE were analysed by liquid chromatography coupled with high-resolution mass spectrometry for both the quantitation of representative phenolic compounds (hesperidin, naringin, p-coumaric acid, caffeic acid, and gallic acid) and for the further screening of other compounds of interest (other phenolic compounds, fatty acids, etc.). For this purpose, we employed a Bruker ELUTE UHPLC (Bruker Daltonics, Bremen, Germany) device connected to a hybrid triple quadrupole/TOF of ion mobility (TimsTOF, Q-TOF) and equipped with an ESI source operating in a positive and negative mode from Bruker Daltonics (Bremen, Germany).

The stationary phase, chromatographic conditions, and ESI source parameters were reported by Sánchez-Vallejo et al. [32]. Acquisition for quantitation was performed with the bbCID (broadband collision induced dissociation) mode and data were processed with the programme TASQ Client 2.1 (Bruker Daltonics). For the identification of other compounds of interest, the acquisition was performed by the autoMSMS mode and data treatment was performed with Metaboscape 6.0.2 (Bruker Daltonics) and open libraries (EU Mass Bank), together with those administered by the equipment vendor for plant metabolites.

OPD extracts were obtained, as described in Section 2.2, by being diluted with methanol, centrifuged (5 min, 10,000 rpm) to remove particles, and then 100 µL of the supernatant was transferred to LC vials with an insert and analysed. Calibration curves were prepared in methanol at concentrations in the ranges of 0.1–1 mg/L for p-coumaric acid, 0.1–0.5 mg/L for caffeic and gallic acids, and 2–25 mg/L for hesperidin and naringin. For the screening of other compounds of interest, identification criteria were set on the basis of exact mass (≤10 ppm), isotopic pattern fit (mSigma ≤ 200), and MS/MS fragmentation score (>900).

### 2.5. In Silico Studies

A set of protein targets relevant to skin health and ageing was identified and the crystal structures were downloaded from the Protein Data Bank (PDB): 2TCL (catalytic domain of fibroblast collagenase), 1ELB (elastase), 4I5I (catalytic domain of NAD-dependent deacetylase sirtuin-1), and 1VJY (the growth factor beta receptor type). All selected protein targets were prepared by removing water molecules and adding missing hydrogen atoms. The co-crystalized ligands in the binding site were removed from structures prior to docking and their centre of mass was used to position a box with a size of 20 Å × 20 Å × 20 Å. The docking of two selected components was conducted using Gold v. 2024.1.0 software and the DockThor web server platform, both using genetic algorithms to generate ligand poses. The docking performed by GOLD software was conducted with GA run for each compound. In a single GA run, 125,000 operations were performed, with the rest of the settings set to autoscale level 2. GoldScore was selected for a scoring function which was used to rank the docking positions of the molecules. The genetic algorithm parameters for DockThor were set to its default values: number of evaluations = 500,000, population size = 750, number of runs = 12, and seed at run #1 = −1985.

### 2.6. Cell Culture

A commercially available rat hepatoma H4IIE cell line was purchased from the American Type Culture Collection (Manassas, VA, USA) (ATCC cat. no. CRL-1548). The cells were cultured and maintained in minimum essential medium (MEM) supplemented with 10% foetal bovine serum (FBS), 1% penicillin/streptomycin (100 U/mL penicillin and 100 µg/mL streptomycin), and 1% L-glutamine in a humidified atmosphere of 5% CO_2_ at 37 °C. The logarithmic growth phase (approximately 70–80% confluence) was used for the following tests. All chemicals were purchased from Capricorn Scientific GmbH (Ebsdorfergrund, Germany).

### 2.7. Preparation of Extract Solution for Treatment

The cells were treated for 24 and 48 h with different concentrations of OPD and its extracts (SWE 3 and PEE 4) ranging from 1 to 300 µg/mL (a stock solution concentration was 3 mg/mL), which were dissolved in fresh culture medium MEM and filtered two times through a bacteriological filter (JCAS022025K, Filter-lab CA syringe filter, 0.22 µm, Filtros Anoia, Barcelona, Spain) to reduce the risk of the contamination of extract solutions. Control cells were treated with fresh medium.

### 2.8. H4IIE Cell Viability Measurement

Cell viability was determined using the MTT [3-(4, 5-dimethylthiazol-2-yl)-2, 5-diphenyltetrazoli-um bromide], as described earlier [33]. H4IIE cells were seeded into a 96-well plate at a density of 2 × 10^4^ cells/well and cultured overnight. Different concentrations of SWE 3 and PEE 4 extracts (0–300 µg/mL) were added and incubated for 24 and 48 h. Following the treatment, 20 μL of the MTT solution (5 mg/mL in MEM) was added and further incubated for 3 h at 37 °C. The insoluble blue-coloured formazan product was dissolved with 100 μL of 0.04 M HCl/isopropanol and incubated at room temperature for 10 min. Absorbance was measured using a 96-well plate reader (Multiscan MCC340, Labsystems, Helsinki, Finland) at a wavelength of 540/690 nm. Measurements were performed two times in quadruplicate. The cell viability was calculated as a percentage using the underlying equation:

$$\mathrm{Cell}\;\mathrm{viability}\;(\%)=\frac{\mathrm{the}\;\mathrm{absorbance}\;\mathrm{of}\;\mathrm{viable}\;\mathrm{cells}\;\mathrm{per}\;\mathrm{well}}{\mathrm{the}\;\mathrm{mean}\;\mathrm{absorbance}\;\mathrm{of}\;\mathrm{the}\;\mathrm{viable}\;\mathrm{cells}\;\mathrm{in}\;\mathrm{control}\;\mathrm{wells}}\times 100\%$$

### 2.9. Morphological Observation

H4IIE cells were plated into six-well plates at a density of 1.5 × 10^4^ cells/well and cultured overnight. Then, the cells were exposed to treatment for 24 and 48 h. The morphology and shape of the H4IIE cells were observed and photographed under an inverted phase contrast microscope (Leica DMIL, LED microscope, Wetzlar, Germany) with the camera Leica MC 190 at 200× magnification. Analysis was performed in a population of 1000 cells.

### 2.10. Colony Formation Assay (Long-Term Cell Survival Assay)

Cells (2 × 10^3^ cells/well) were initially grown in six-well plates overnight. The cells were then treated with prepared extract solutions with different concentrations (0–300 µg/mL) for 24 h at 37 °C. Subsequently, the treatment was discarded, the cells were washed with PBS and a fresh complete medium was added. The medium was changed twice a week. After 14 days, colonies were fixed with 100% methanol for 20 min at room temperature and stained with 0.5% crystal violet solution [34]. Due to a large number of colonies, the results were interpreted only qualitatively using a stereomicroscope (OZR 564, KERN Optics, Kern & Sohn GmbH, Balingen-Frommern, Germany).

### 2.11. Statistical Analysis

Results related to EY, TPC, and LC-ESI-MS/MS quantification were presented as mean ± standard deviation (SD). Statistical differences between samples were assessed using one-way analysis of variance (ANOVA), followed by Tukey’s post hoc test, with significance considered at *p* < 0.05.

For cytotoxicity assays, the results were also presented as mean ± SD. Variations between groups were statistically analysed in SPSS v26.0 (IBM Corp., Chicago, IL, USA) using one-way ANOVA with Tukey’s post hoc test. The graphical presentation of the results was carried out with GraphPad Prism software package version 7.05 (San Diego, CA, USA). Statistical significance was considered as follows: * *p* < 0.05 and # *p* < 0.001.

## 3. Results and Discussion

### 3.1. Extraction Yield and Total Phenolic Content of Obtained OPD Extracts

Various factors are known to influence the presence of biologically active substances in orange peel, including cultivation practices, climatic conditions, morphology, soil composition, and variety, but also the type and parameters of extraction. As a strategy for OPD valorisation and the recovery of biologically active compounds from this kind of waste material, two green techniques (SWE and PEE) were applied under different process set-ups in order to evaluate the impact of process parameters and to define the extraction protocol which would result in the production of OPD extracts of the highest quality. Extraction conditions and results are given in Table 1.

The EY values, obtained in the case of the SWE of OPD, ranged from 30.03 to 66.67%, showing a slight dependence of the yield on the applied process parameters. EY increased as the temperature increased from 120 °C to 140 °C, then a slight decreasing trend began with a further increase in the applied temperature. The maximum EY value of SWE extracts (66.67 ± 0.45%) was obtained at a temperature of 140 °C, an extraction time of 15 min, and a process pressure of 20 bar, while the lowest yield (30.03 ± 0.64%) was obtained at the highest temperature and was 2.2 times lower compared to the maximum. In the case of the PEE of OPD, the yield increase showed a slightly slower trend compared to SWE, and EY grew and reached the maximum yield at a temperature of 160 °C (72.60 ± 2.07%), after which the yield also began to decrease, and it was much more noticeable in the interval between 200 °C and 220 °C. If we compare these two techniques in terms of yield, it can be concluded that the maximum yield with PEE was achieved at a slightly higher temperature and that it was 1.09 times higher than the maximum yield achieved using SWE.

Thanks to a series of studies conducted so far, it is now well established that high-value polyphenolic compounds in citrus fruits play a very important role both in improving human health and delaying/preventing the onset of a large number of chronic diseases [35]. They have shown exceptional activity in removing free radicals to protect fruits and vegetables from infection by various pathogens, preventing photo-oxidative damage by UV radiation, etc. [36]. Polyphenolic compounds in citrus peels possess a wide range of bioactivities that improve human health, including anti-inflammatory, anti-allergic, antiproliferative, antioxidant, antiviral, neuroprotective, anti-carcinogenic, and antimicrobial properties that protect against oxidative stress-related diseases [35,37]. It was previously demonstrated by Guimarães et al. [38] that citrus peels contained higher levels of TPC than other parts of citrus fruits.

In this work, TPC in different SWE OPD extracts ranged from 13.83 to 36.52 mg GAE/g dw. A significant increase in the phenol content was observed from 120 to 180 °C reaching a maximum at 180 °C (36.52 ± 0.30 mg GAE/g dw). With a further increase in temperature, the phenol content decreased, and the SWE OPD extract obtained at the highest temperature of 220 °C had 1.02 times lower phenol content compared to the highest value, while the lowest TPC was obtained at the lowest temperature of 120 °C and was 2.64 times lower compared to the highest value. The increase in TPC originated from the increased solubility of this group of compounds in subcritical water due to the increase in process temperature. Water at room temperature has a relatively high dielectric constant, which decreases due to the application of high temperatures and therefore increases the extraction power of water. Moreover, the application of high temperatures favours the kinetics of mass transfer by disrupting the interaction of the matrix and the analyte, especially dipole–dipole forces and hydrogen bonding, which greatly facilitates the initial desorption of the analyte from the sample matrix. Also, there is an acceleration of diffusion and a change in solubility [39]. However, higher temperatures, in this case higher than 180 °C, lead to the decomposition of phenolics. Regarding PEE, the TPC ranged from 16.59 to 70.56 mg GAE/g dw. In relation to SWE, in this type of extraction, TPC constantly increased with the increase in temperature, and the maximum was obtained at 220 °C (70.56 ± 5.85 mg GAE/g dw). The obtained TPC was almost 2 times higher compared to the maximum TPC obtained using subcritical water. The increase in TPC with the applied temperature was recorded by Benito-Román et al. [40], who isolated the highest TPC from onion skin waste using SWE at the highest applied temperature (180 °C).

In a study conducted by Brezo-Borjan et al. [41], the influence of temperature (120–200 °C) and time (5–60 min) on TPC from orange peel obtained using SWE was investigated. The TPC ranged from 27.58 mg GAE/g dw to 45.45 mg GAE/g dw, which is in agreement with the results obtained in this study.

### 3.2. LC-ESI-MS/MS Profiling of Liquid OPD Extracts

The LC-ESI-MS/MS quantitative analysis of OPD extracts, obtained in SWE and PEE, confirmed the presence of all the targeted bioactive compounds, namely hesperidin and naringin, followed by p-coumaric, gallic, and caffeic acids. According to the literature, the two most dominant flavonoids in the fresh and dried orange by-products were hesperidin and narirutin [42]. This was also confirmed in our previous study where ultrasound-assisted extraction was applied for the recovery of hesperidin and narirutin, as well as naringin and rutin, and the results showed that these compounds could be extracted in significant amounts from OPD [43]. M’hiri et al. [44] identified 10 phenolic compounds in ethanol extracts of Maltese orange peel obtained by different methods, namely flavanones (eriocitrin, narirutin, naringin, hesperidin, neohesperidin, didimine) and polymethoxylated flavones (sinensetin, hexamethoxyflavone, nobiloxyflavone). Also, hesperidin was one of the most dominant compounds in the study conducted by Londono-Londono et al. [45].

As already mentioned, the most common flavonoid in orange peel is hesperidin. According to the results, the same flavonoid is the common flavonoid of OPD, which is meaningful, as OPD is produced during the orange peel processing; therefore, it is the same natural material. Based on Table 2, it is obvious that hesperidin is the most dominant flavonoid in all tested OPD extracts. Naringin was present as the second most dominant compound, while phenolic acids were identified as supporting elements for which the concentration was quite low.

According to the literature, Lachos-Perez et al. [46] investigated the possibility of using SWE to obtain high-added-value products from orange peel (OP) as a first step in a biorefinery concept. Hesperidin and narirutin were identified as the most dominant flavonoids. The yield of hesperidin ranged from 9.82 ± 0.01 to 22.99 ± 0.7 mg/g OP, while the yield of narirutin was significantly lower (1.9 ± 0.2 mg/g OP), reaching the highest value at 150 °C using a solvent flow of 10 mL/min. A study by Barrales et al. [47] aimed to investigate and compare the possibility of extracting phenolic compounds from the orange peel with and without supercritical carbon dioxide pretreatment using three different techniques: PLE, UAE, and Soxhlet extraction. Among the applied extraction methods, PLE achieved the highest yields of glycosylated flavonoids such as hesperidin, naringin, and narirutin. Among the identified flavonoids, hesperidin was the most abundant, which corresponds to the typical composition of flavonoids in citrus peel [48]. The PLE solvents were absolute ethanol and mixtures of ethanol and water (75% and 50% ethanol, *v*/*v*) at temperatures of 45, 55, and 65 °C and a pressure of 10 MPa. The best PLE conditions for the isolation of hesperidin were the use of 75% ethanol at 65 °C; however, when this concentration was compared to the extraction conducted using 50% ethanol, it could be concluded that the concentration of hesperidin was not significantly different. Hwang et al. [49] proved in their research that the extraction efficiency of narirutin and hesperidin from *Citrus unshiu* peel was improved by the pulsed electric field (PEF). Therefore, the samples were first treated with a PEF of 3 kV/cm for 60 and 120 s. After that, SWE was performed at temperatures ranging from 110 to 190 °C for 3 to 15 min. The maximum concentration of hesperidin was 46.96 mg/g dry peel after PEF treatment for 120 s, with a temperature of 150 °C for 15 min, while the highest concentration of narirutin was 8.7 mg/g dry peel after PEF treatment for 120 s, with a temperature of 190 °C for 5 min. Narirutin and hesperidin increased with the duration of PEF treatment. PEF increased the amount of extracted hesperidin by 22.1% and narirutin by 33.6%, respectively.

The concentration of hesperidin in the liquid OPD extracts obtained in SWE ranged from 0.09 ± 0.10 to 662.82 ± 22.11 mg/L depending on the applied temperature. The obtained values are in accordance with the previously mentioned literature. The highest concentration was obtained at a temperature of 160 °C, and after that, there was a significant decrease in the concentration of this high-value constituent. This is consistent with research conducted by Lachos-Perez et al. [46] who found that SWE degradation of hesperidin from defatted orange peel occurred at temperatures greater than 160 °C. If the process was carried out using 50% (*v*/*v*) ethanol as a solvent, then liquid OPD extracts with a slightly lower hesperidin concentration (5.81 ± 4.99–491.77 ± 28.89 mg/L) could be obtained. However, since the concentration of TP was significantly higher in this case, a detailed analysis of the obtained samples should be carried out, due to the possibility of creating new antioxidants.

Another dominant flavonoid, naringin, known for its antioxidant abilities [50], ranged in liquid SWE OPD extracts between 0.14 ± 0.20 and 62.37 ± 2.05 mg/L, with the highest concentration obtained at the lowest temperature (120 °C). In the second investigated case, the concentration of this compound was in the range of 0.66 ± 0.26–51.49 ± 3.57 mg/L, where the highest concentration was obtained at a significantly higher temperature compared to SWE extracts (180 °C). As in the case of hesperidin, the degradation of the naringin was noticed in the case of both applied processes. For SWE, the rapid degradation of naringin started above 160 °C, and this compound was almost all degraded at 220 °C. For PEE, the degradation was not so rapid; at 200 °C, the concentration decreased to approximately 50% of the highest obtained in PEE, but then at 220 °C was almost completely degraded. The same pattern of slower degradation was noticed for hesperidin.

The other targeted compounds (phenolic acids, namely p-coumaric, caffeic, and gallic acids) were detected at much lower concentrations in all OPD liquid extracts, either obtained by SWE or by PEE. These acids were also identified by Castro-Vázquez et al. [51] in their research as being compounds present in orange peel.

To the best of our knowledge, the comprehensive elucidation of compounds of interest in this type of extract was performed for the first time and carried out by LC-ESI-MS/MS. In liquid SWE OPD extracts, a wide variety of compounds—a total of 101 compounds—were identified, while in the case of PEE OPD extracts, even more—131 compounds—were identified. In total, 153 different compounds were tentatively identified. Results are shown in Table S1. The samples are marked with SWE and PEE depending on the type of extraction by which they were obtained, as well as numbers from 1 to 6 depending on the applied process temperature (from the lowest to the highest). Together with the previously quantified hesperidin (hesperetin-7-O-rutinoside), its unconjugated form (hesperetin) was also detected in all the SWE and PPE extracts. The flavanone glycosides derived from naringin, namely naringenin-7-O-glucoside and naringenin-7-O-rutinoside or narirutin, were also detected, with the latter only being present in PEE extracts. Other less abundant flavonoids were also present in all the extracts, such as luteolin, a recognised potent antioxidant, and its derivatives (diosmin, 6-methoxyluteolin, and luteolin-6-C-glucoside, the latter only being present in SWE extracts). Further tentatively identified minor flavonoids included rutin, limocitrin, isosakuranetin, and catechin, among others. Other phenolic compound classes with antioxidant potential were also detected, such as hydroxyferulic acid, homovanillic acid, hydroxycinnamic acids (hydroxyferulic acid, 7,8-dihydroxycoumarin, etc.), and vanillin, among many others. Finally, other common plant metabolites were also identified, such as organic acids (malic acid, tartaric acid, citric acid, succinic acid, etc.), saccharides (D-galactose, D-ribose, etc.), and fatty acids and derivatives (palmitic acid, FA 18:2 + 1O, FA 18:2 + 2O, etc.).

Although SWE gave slightly higher concentrations of both hesperidin and naringin, this technique was more susceptible to spoilage and contamination by various microorganisms due to the nature of water extracts. In contrast, PEE extracts obtained using 50% ethanol as a solvent represent a significantly safer and more stable medium in terms of contamination. Furthermore, the results for TPC were significantly higher. The complex mixture of compounds with potential antioxidant properties tentatively identified by wide MS screening and possible synergistic effects could explain the difference. In fact, as mentioned before, a higher number of compounds of interest were detected in PEE extracts.

### 3.3. In Silico Evaluation of Skin Anti-Ageing Potential

The molecular docking of hesperidin and naringin against several protein targets implicated in skin ageing was performed using the GOLD software and binding affinities were predicted using the DockThor rescoring function (Table 3). The higher GOLDScore fitness values and more negative DockThor binding affinities (kcal/mol) indicated stronger predicted binding to the targets. This consensus of the molecular docking results suggested that hesperidin and naringin have the potential to interact with ageing-related proteins including collagenase, elastase [52], sirtuin-1 [53], and growth factor beta receptor [54]. Across the targets, hesperidin exhibited slightly higher predicted binding affinity based on the GOLDScore fits and DockThor scores. The consensus of the two methods suggested that the strongest interactions of two flavonoids were predicted with protein sirtuin-1 and growth factor beta receptor. For example, the flavonoidic backbone of the hesperidin could fit tightly into the hydrophobic cleft of the growth factor beta receptor and the glycoside moiety had additional affinity with the hydrophilic surface (Figure S1a), providing selectivity through the interaction with the Lys232 of the selectivity pocket [55] (Figure S1b). Similarly, naringin was predicted to adopt a comparable orientation and interaction pattern (Figure S2), suggesting its glycoside moiety may have contributed to selective binding akin to hesperidin.

Overall, the molecular modelling provided evidence that hesperidin and naringin can favourably interact with multiple biochemical targets linked to skin ageing, with hesperidin showing a slight edge over naringin. The findings provided insights into the molecular mechanisms by which these citrus flavonoids may mitigate skin ageing and support further investigation into their anti-ageing bioactivities.

While hesperidin and naringin could likely contribute to the bioactivity due to their abundance, the cosmetic benefits may also be derived from not only their synergistic interactions but also from their synergistic interaction with other compounds present in the extract via polypharmacology. Specifically, additional flavonoids like kaempferol [56] and quercetin [57] could provide complementary antioxidant and anti-inflammatory activities while organic acids like citric may enhance the absorption and bioavailability of the primary flavonoids [58] and limonene and menthol may improve skin permeation [59].

The multi-target activities predicted for the flavonoids suggest they may modulate interconnected pathways involved in various ageing phenotypes through network pharmacology effects. Cosmetic formulations incorporating citrus extracts like these, with bioactive profiles optimised based on the relative concentrations of key actives like hesperidin and naringin, may provide more comprehensive anti-ageing benefits by simultaneously supporting extracellular matrix remodelling, collagen production, and cell proliferation. However, some components like essential oils and simple phenols could potentially cause irritation or sensitization at high concentrations [60], while mycotoxins such as citrinin must be monitored and limited to safe levels [61]. Therefore, careful formulation and testing would be needed to fully evaluate synergies and safety, while further studies could explore the extraction conditions to OPD to optimise extracts to have the potential for application in multi-faceted and safe anti-ageing cosmetic products.

### 3.4. In Vitro Evaluation of Antitumor Potential

In recent years, more and more attention has been focused on research whose goal is the evaluation of anticancer bioprospecting on medicinal plants [62,63,64,65]. According to this burning problem, this study investigated the anticancer activity of OPD and different extracts prepared with different extraction methods on rat hepatoma cells.

**Figure 1:**
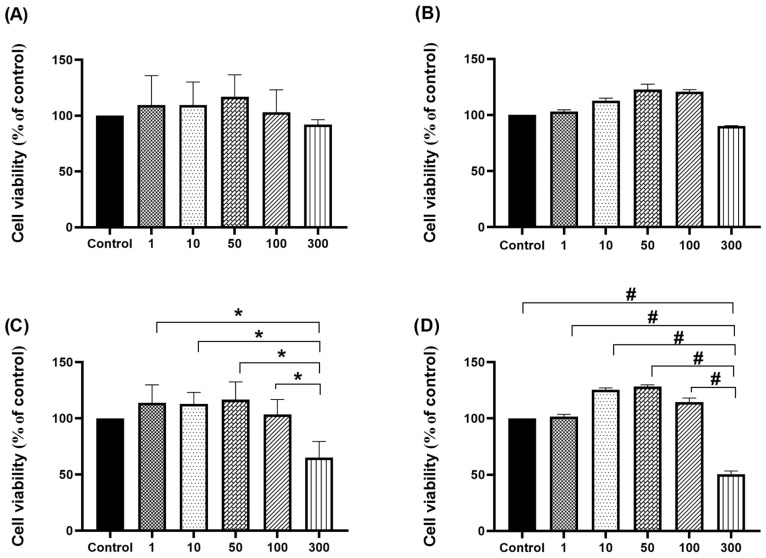
Effect of different concentrations (1 µg/mL, 10 µg/mL, 50 µg/mL, 100 µg/mL, and 300 µg/mL) of SWE 3 (extract obtained with subcritical water extraction procedure) (**A**,**B**) and PEE 4 (extract obtained with the pressurised ethanol extraction procedure) (**C**,**D**) extracts on the viability of H4IIE cells, measured by MTT assay. Cells were incubated with extracts for 24 h (**A**,**C**) and 48 h (**B**,**D**). Treatment with 300 µg/mL of the extract obtained with the pressurised ethanol extraction procedure in both periods significantly decreased the tumour growth rate compared to the other experimental groups (**C**,**D**) and the control (**D**). Results are expressed as viable cells (% of control) ± standard deviation (SD). Differences between groups were analysed where * *p* < 0.05 and # *p* < 0.001.

To measure the viability of H4IIE cells after treatment with SWE 3 and PEE 4 extracts for different periods (24 h and 48 h), an MTT assay was used (Figure 1). After a 24 h incubation with sample extracts from water (SWE 3), there was no significant cytotoxicity at any tested concentrations (Figure 1A). The addition of the highest tested concentration, which was 300 µg/mL, for 48 h exhibited negligible cytotoxicity and a decrease in measured absorbance compared to other groups (survival rate 90%) (Figure 1B). Chen et al. [66] evaluated the influence of the dehydrated powder of the sweet orange peel (*Citrus sinensis* L.) suspended in water (WESP) on the cell viability of human hepatocellular carcinoma (HepG2). After cells were cultured with WESP in different concentrations ranging from 0 to 1000 µg/mL for 24 h, cell viability was determined by an MTT assay. They concluded that cell survival in the presence of all tested concentrations was >85%, so this extract had no cytotoxicity to HepG2 cells, which is consistent with our findings.

On the other hand, after H4IIE cells were cultured in a medium containing 300 µg/mL of PEE 4 extract, this treatment in both periods of time induced a significant decrease in tumour growth compared to other experimental groups (Figure 1C,D) and the control (Figure 1D) (* *p* < 0.05, # *p* < 0.001). The observed high toxicity of this ethanolic extract aligned with the findings reported by Banjerdpongchai et al. [67], who investigated the effect of bioflavonoids from citrus seeds against the HepG2 cell line. In this study, cells were treated with crude ethanolic extract from *Citrus* seeds and the commercially available active compounds hesperidin and naringin at various concentrations for 24 h. Results showed that IC_50_ values of more than 200 mg/μL from both the ethanolic crude extract of *Citrus* seed and the bioflavonoids from *Citrus* seeds had antitumor potential. The cytotoxic effect was attributed to active flavanone glycosides, hesperidin [68], and naringin [69]. These compounds demonstrated various biological activities through the regulation of several cellular signalling pathways, such as antioxidant [70], anti-inflammatory [71], and anticancer properties [72,73,74,75,76]. Studies have shown that a possible explanation is in the inhibition of cellular proliferation through different mechanisms such as growth suppression and development via apoptosis by inducing both extrinsic and intrinsic pathways [75,77,78,79], and cell cycle arrest at the G0/G1 phases in malignant cells [80].

**Figure 2:**
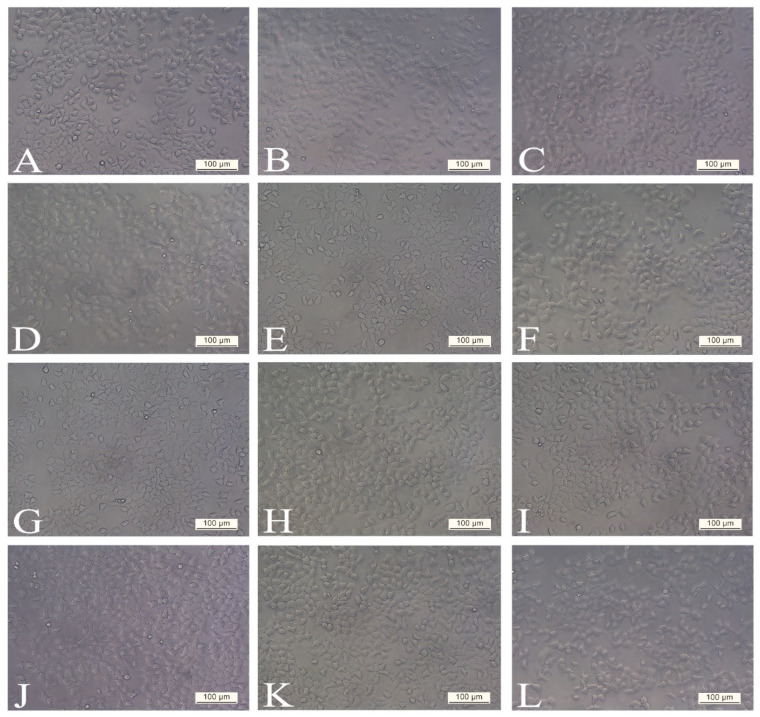
Morphology of the H4IIE cell line exposed to various concentrations of SWE 3 (extract obtained with a subcritical water extraction procedure): (**A**,**G**) control (non-treated), (**B**,**H**) treated with 1 µg/mL, (**C**,**I**) treated with 10 µg/mL, (**D**,**J**) treated with 50 µg/mL, (**E**,**K**) treated with 100 µg/mL, (**F**,**L**) treated with 300 µg/mL. Cellular morphological changes were analysed with phase contrast in different periods (**A**–**F**) after 24 h and (**G**–**L**) after 48 h. There were no observable differences in cell morphology and structure at both periods compared to the control group of cells. All photographs were taken at 200× magnification. Scale bars represent 100 µm.

**Figure 3:**
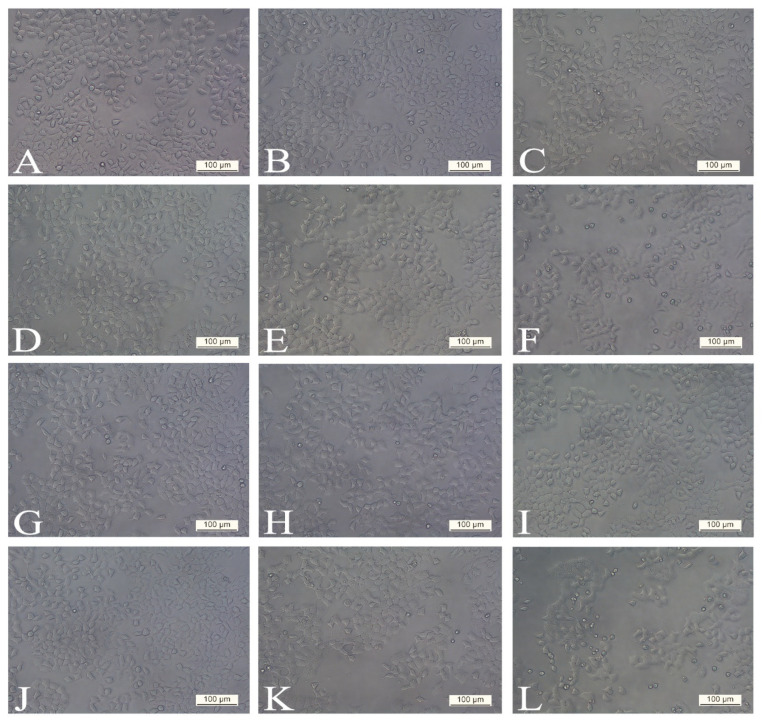
Morphology of the H4IIE cell line exposed to various concentrations of PEE 4 (extract obtained with the pressurised ethanol extraction procedure): (**A**,**G**) control (non-treated), (**B**,**H**) treated with 1 µg/mL, (**C**,**I**) treated with 10 µg/mL, (**D**,**J**) treated with 50 µg/mL, (**E**,**K**) treated with 100 µg/mL, (**F**,**L**) treated with 300 µg/mL. Cellular morphological changes were analysed with phase contrast in different periods: (**A**–**F**) after 24 h and (**G**–**L**) after 48 h. The extract obtained with the pressurised ethanol extraction procedure at 300 µg/mL concentration caused classic apoptotic cell death (rounded and smaller cells with nuclear disintegration and chromatin condensation). All photographs were taken at 200× magnification. Scale bars represent 100 µm.

Evaluation of morphological characteristics under an inverted microscope with phase contrast showed that there were no observable differences in cell morphology and structure at both 24 h and 48 h post-treatment with SWE 3 extract (1–300 µg/mL) compared to the control group of cells (Figure 2). Also, treatment with concentrations ranging from 1 to 100 µg/mL of PEE 4 extract produced no significant effect on the H4IIE cell morphology and cell death (Figure 3B–E,H–K), which was in agreement with the results of cell viability and showed that the tested extracts in the noted concentrations exhibited no cytotoxicity effect.

When cells were treated with a dose of 300 µg/mL of PEE 4 after 24 h (Figure 3F) and 48 h (Figure 3L), significant morphological changes in cell structure were observed in comparison to the control group (Figure 3A,G). Cells were more spherical and had smaller dimensions. After 48 h, extensive damage in the cellular structure could be seen. Nuclear disintegration and chromatin condensation of the treated cells was observed. In addition, the number of dead rounded cells and their debris was significantly higher 48 h post-treatment. These results indicate that the extract PEE 4 causes classic apoptotic cell death.

**Figure 4:**
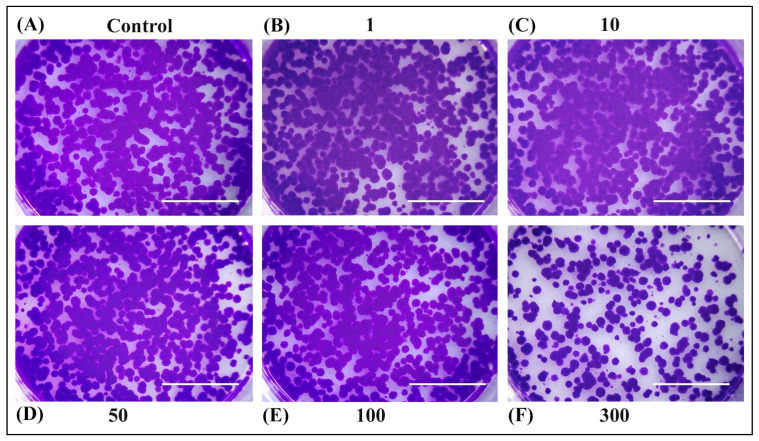
Rat hepatoma cells H4IIE were seeded in six-well plates (2000 cells/well) for long-term survival assay. After 24 h, the culture medium was replaced with (**A**) fresh medium or medium containing (**B**) 1 µg/mL, (**C**) 10 µg/mL, (**D**) 50 µg/mL, (**E**) 100 µg/mL, and (**F**) 300 µg/mL of SWE 3 extract (obtained with a subcritical water extraction procedure). Then, the cells were incubated for another 24 h. The culture medium was changed every three days for two weeks. Cells were fixed with methanol and stained with crystal violet. A 300 µg/mL concentration exerted a strong inhibitory effect on cell survival. Scale bars represent 1 cm.

**Figure 5:**
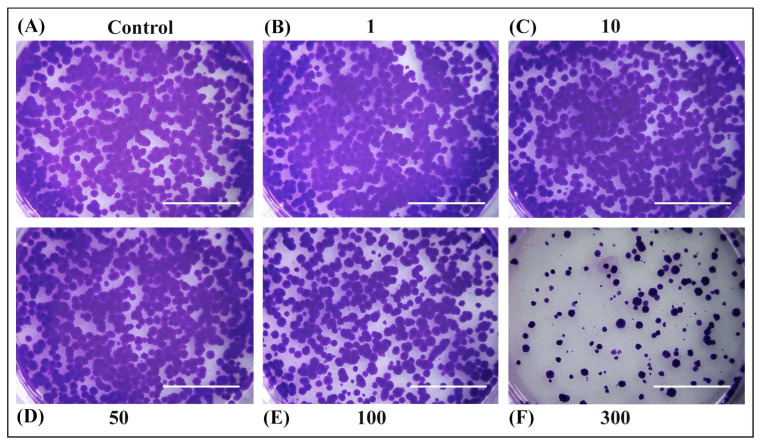
Rat hepatoma cells H4IIE were seeded in six-well plates (2000 cells/well) for long-term survival assay. After 24 h, the culture medium was replaced with (**A**) fresh medium, or medium containing (**B**) 1 µg/mL, (**C**) 10 µg/mL, (**D**) 50 µg/mL, (**E**) 100 µg/mL, and (**F**) 300 µg/mL of PEE 4 extract (obtained with the pressurised ethanol extraction procedure). Then, the cells were incubated for another 24 h. The culture medium was changed every three days for two weeks. Cells were fixed with methanol and stained with crystal violet. The higher concentrations of 100 µg/mL exerted a strong inhibitory effect on cell survival. Scale bars represent 1 cm.

The ability of OPD extracts to inhibit cell growth was also determined by colony formation assay (Figure 4 and Figure 5). No significant differences were observed in long-term cell survival upon treatment with extracts SWE 3 and PEE 4 in concentrations of 1–50 µg/mL compared to the control. However, higher concentrations of 100 µg/mL of PEE 4 extract (Figure 5E) or 300 µg/mL of SWE 3 and PEE 4 exerted a strong inhibitory effect on cell survival (Figure 4F and Figure 5F).

These results from an in vitro study on rat hepatoma cells suggested that OPD extracts have antitumor potential. The quote activity of the ethanolic extract showed a higher potential compared to the water extract of orange peel. Similar results were reported by Gavamukulya et al. [81]. Analysis of the ethanolic and water leaf extracts of *A. muricata* leaves indicated that water extracts showed no effect throughout the range of tested concentrations (0, 250, 500, 750, 1000, and 1250 μg/mL) on the cell proliferation of human breast cancer cell lines MDA and SKBR3. However, the ethanolic extract had a very high cytotoxic potential with an IC_50_ of 248.77 μg/mL and 202.33 μg/mL against MDA and SKBR3 cell lines, respectively. A possible explanation of our results is that the extracts contain other active biological compounds in addition to the isolated ones, but the methods for their identification are not readily available. Some of these compounds may have been present in very high quantities in the ethanolic extract, or the concentrations were lower or even absent in the water extract, leading to the discrepancy of anticancer potential. Lastly, more experimental in vitro studies are needed to elucidate the root cause of this difference.

## 4. Conclusions

In this study, different process parameters of green extraction methods, namely subcritical water extraction and ethanol extraction under pressure, were investigated, in order to determine the most adequate ones that ensured high-quality OPD extracts and maximum raw material exploitation without negative impacts on the environment. It was concluded that lower subcritical water extraction temperatures were more suitable for OPD valorisation because they provided a higher content of dominant antioxidants (hesperidin and naringin). However, pressurised ethanol extraction proved to be a more suitable method in terms of isolating TPC, as well as providing a higher extraction yield and a higher number of identified compounds of interest. The present findings from an in vitro study on rat hepatoma cell culture confirmed that PEE OPD extracts exerted stronger antitumor potential than SWE OPD extracts. Also, according to the in silico test results, and thanks to the bioactivity of the dominant compounds hesperidin and naringin, OPD extracts incorporated into cosmetic formulations could be beneficial against ageing by simultaneously supporting extracellular matrix remodelling, collagen production, and cell proliferation. These research results make a significant contribution to the concept of the circular economy considering that they propose sustainable solutions that include the reduction in waste/by-products from the filter tea industry, i.e., food, as well as the exploitation of OPD through making maximum use of natural resources.

## Figures and Tables

**Table 1:** Extraction yield (EY) and total phenolic content (TPC) obtained using pressurised liquid extractions (PLEs).

Samples	Pressure [bar]	Temperature [°C]	EY [%]	TPC [mg GAE/g Dry Weight]
SWE orange peel 1	20	120	60.22 ± 0.86 ^a, 2–4^	13.83 ± 0.001 ^b, 6^
SWE orange peel 2	20	140	66.67 ± 0.45 ^a, 1–2^	20.51 ± 2.85 ^b, 5^
SWE orange peel 3	20	160	64.43 ± 1.50 ^a, 1–3^	32.39 ± 2.85 ^a, 4^
SWE orange peel 4	20	180	52.37 ± 0.76 ^ab, 4–5^	36.52 ± 0.30 ^a, 4^
SWE orange peel 5	20	200	42.07 ± 2.62 ^bc, 5–6^	35.99 ± 3.15 ^a, 4^
SWE orange peel 6	20	220	30.03 ± 0.64 ^c, 7^	35.67 ± 1.50 ^a, 4^
PEE orange peel 1	20	120	54.48 ± 0.90 ^D, 3–4^	16.59 ± 1.20 ^E, 5–6^
PEE orange peel 2	20	140	63.03 ± 1.36 ^BC, 1–4^	18.92 ± 2.40 ^E, 5–6^
PEE orange peel 3	20	160	72.60 ± 2.07 ^A, 1^	32.17 ± 1.05 ^D, 4^
PEE orange peel 4	20	180	66.52 ± 0.77 ^B, 1–2^	46.07 ± 2.10 ^C, 3^
PEE orange peel 5	20	200	60.70 ± 0.22 ^C, 2–4^	56.88 ± 3.60 ^B, 2^
PEE orange peel 6	20	220	38.93 ± 1.43 ^E, 6–7^	70.56 ± 5.85 ^A, 1^

Different letters within a column indicate significant differences between samples at *p* < 0.05: lowercase letters are related to SWE extracts, uppercase letters are associated with PEE extracts, and numbers represent the difference between SWE and PEE extracts. SWE—subcritical water extraction; PEE—pressurised ethanol extraction.

**Table 2:** Compounds quantified by liquid chromatography–high resolution tandem mass spectrometry (LC-ESI-MS/MS) in orange peel dust (OPD) extracts.

Samples	Temperature [°C]	Hesperidin [mg/L]	Naringin [mg/L]	p-Coumaric Acid [mg/L]	Gallic Acid [mg/L]	Caffeic Acid [mg/L]
SWE orange peel 1	120	180.28 ± 20.05 ^c, 5^	62.37 ± 2.05 ^a, 1^	0.32 ± 0.01 ^b, 3–4^	0.02 ± 0.001 ^d, 5^	0.07 ± 0.001 ^b, 4–5^
SWE orange peel 2	140	401.02 ± 21.21 ^b, 3–4^	57.65 ± 8.41 ^b, 1–3^	0.31 ± 0.06 ^b, 3–4^	0.05 ± 0.01 ^c, 3–4^	0.18 ± 0.01 ^a, 2–3^
SWE orange peel 3	160	662.82 ± 22.11 ^a, 1^	59.99 ± 2.65 ^ab, 1–2^	0.40 ± 0.05 ^a, 3^	0.12 ± 0.001 ^a, 1^	0.19 ± 0.01 ^a, 1–2^
SWE orange peel 4	180	173.43 ± 13.80 ^c, 5^	12.17 ± 1.09 ^c, 7^	0.21 ± 0.16 ^c, 4^	0.12 ± 0.001 ^a, 1^	0.06 ± 0.001 ^b, 4–6^
SWE orange peel 5	200	6.39 ± 8.99 ^d, 6^	0.83 ± 0.42 ^d, 8^	0.04 ± 0.03 ^d, 5^	0.12 ± 0.001 ^a, 1^	0.04 ± 0.02 ^c, 6–7^
SWE orange peel 6	220	0.09 ± 0.10 ^d, 6^	0.14 ± 0.20 ^d, 8^	0.05 ± 0.05 ^d, 5^	0.07 ± 0.01 ^b, 2^	-
PEE orange peel 1	120	366.42 ± 12.75 ^BC, 3–4^	49.49 ± 1.07 ^AB, 3–4^	0.38 ± 0.07 ^C, 3^	0.01 ± 0.001 ^C, 5^	0.03 ± 0.01 ^E, 7^
PEE orange peel 2	140	325.50 ± 40.26 ^C, 4^	46.56 ± 3.61 ^AB, 4–5^	0.76 ± 0.05 ^B, 2^	0.02 ± 0.01 ^C, 5^	0.08 ± 0.01 ^C, 4^
PEE orange peel 3	160	441.76 ± 58.88 ^AB, 2–3^	51.49 ± 3.57 ^A, 2–4^	1.19 ± 0.05 ^A, 1^	0.04 ± 0.01 ^B, 4^	0.21 ± 0.01 ^A, 1^
PEE orange peel 4	180	491.77 ± 28.89 ^A, 2^	40.56 ± 2.38 ^B, 5^	0.66 ± 0.09 ^B, 2^	0.07 ± 0.001 ^A, 2^	0.16 ± 0.02 ^B, 3^
PEE orange peel 5	200	323.84 ± 28.72 ^C, 4^	25.15 ± 3.04 ^C, 6^	0.29 ± 0.10 ^C, 3–4^	0.07 ± 0.001 ^A, 2^	0.05 ± 0.02 ^DE, 5–7^
PEE orange peel 6	220	5.81 ± 4.99 ^D, 6^	0.66 ± 0.26 ^D, 8^	0.04 ± 0.00 ^D, 5^	0.06 ± 0.01 ^A, 2–3^	0.06 ± 0.03 ^CD, 4–6^

Different letters within a column indicate significant differences between samples at *p* < 0.05: lowercase letters are related to SWE extracts, uppercase letters are associated with PEE extracts, and numbers represent the difference between SWE and PEE extracts. SWE—subcritical water extraction; PEE—pressurised ethanol extraction.

**Table 3:** In silico prediction of binding interactions between most abundant flavonoids in the orange peel extract and selected skin ageing-associated proteins. The molecular docking was conducted using Gold software and the DockThor web platform.

Component	Hesperidin		Naringin	
Protein Target	GoldScore Fitness Score	DockThor Docking Score (kcal/mol)	Gold Fitness Score	DockThor Docking Score (kcal/mol)
Fibroblast collagenase	72.7	−0.8	66.2	−0.7
Elastase	63.8	−9.2	57.2	−8.7
Sirtuin-1	88.4	−10.2	89.6	−10.1
Growth factor beta receptor	86.5	−10.0	68.0	−10.2

## Data Availability

The data that support the findings of this study are available upon request from the corresponding author.

## Supplementary Materials

The following supporting information can be downloaded at: https://www.mdpi.com/article/10.3390/antiox14060638/s1. Table S1: LC-ESI-MS/MS qualitative analysis of liquid OPD extracts; Figure S1: A representative docked pose of hesperidin into the binding site of growth factor beta receptor (surface coloured according to hydrophobicity) shown as (a) a three-dimensional representation of a ligand (thick green sticks) and (b) a two-dimensional protein ligand plot; Figure S2: A representative docked pose of naringin into the binding site of growth factor beta receptor (surface coloured according to hydrophobicity) shown as (a) a three-dimensional representation of a ligand (thick green sticks) and (b) a two-dimensional protein ligand plot.

## References

[1] Colegate S.M., Molyneux R.J. (2007). Bioactive Natural Products: Detection, Isolation, and Structural Determination.

[2] Vidović S., Vladić J., Nastić N., Jokić S., Muthukumarappan K., Knoerzer K. (2021). Subcritical and supercritical extraction in food by-product and food waste valorization. Innovative Food Processing Technologies: A Comprehensive Review.

[3] Cheng Y., Xue F., Yu S., Du S., Yang Y. (2021). Subcritical Water Extraction of Natural Products. Molecules.

[4] Vladić J., Jakovljević M., Molnar M., Vidović S., Tomić M., Drinić Z., Jokić S. (2020). Valorization of Yarrow (*Achillea millefolium* L.) by-Product through Application of Subcritical Water Extraction. Molecules.

[5] Vidović S., Nastić N., Gavarić A., Cindrić M., Vladić J. (2019). Development of green extraction process to produce antioxidant-rich extracts from purple coneflower. Sep. Sci. Technol..

[6] Vladić J., Canli O., Pavlić B., Zeković Z., Vidović S., Kaplan M. (2017). Optimization of Satureja Montana Subcritical Water Extraction Process and Chemical Characterization of Volatile Fraction of Extracts. J. Supercrit. Fluids.

[7] Lachos-Perez D., Brown A.B., Mudhoo A., Timko M.T., Rostagno M.A., Forster-Carneiro T. (2017). Applications of Subcritical and Supercritical Water Conditions for Extraction, Hydrolysis, Gasification, and Carbonization of Biomass: A Critical Review. Biofuel Res. J..

[8] Ciftci D., Saldaña M.D.A. (2015). Hydrolysis of Sweet Blue Lupin Hull Using Subcritical Water Technology. Bioresour. Technol..

[9] Munir M.T., Kheirkhah H., Baroutian S., Quek S.Y., Young B.R. (2018). Subcritical Water Extraction of Bioactive Compounds from Waste Onion Skin. J. Clean. Prod..

[10] Monrad J.K., Howard L.R., King J.W., Srinivas K., Mauromoustakos A. (2010). Subcritical Solvent Extraction of Anthocyanins from Dried Red Grape Pomace. J. Agric. Food Chem..

[11] Marcus Y. (2018). Extraction by Subcritical and Supercritical Water, Methanol, Ethanol and Their Mixtures. Separations.

[12] Rahmana Putra N., Nur Rizkiyah D., Idham Z., Abbas Ahmad Zaini M., Azizi Che Yunus M., Hazim Abdul Aziz A. (2023). Optimization and Solubilization of Interest Compounds from Roselle in Subcritical Ethanol Extraction (See). Alex. Eng. J..

[13] Mufari J.R., Rodríguez-Ruiz A.C., Bergesse A.E., Miranda-Villa P.P., Nepote V., Velez A.R. (2021). Bioactive Compounds Extraction from Malted Quinoa Using Water-Ethanol Mixtures under Subcritical Conditions. LWT.

[14] Chiou T.-Y., Neoh T.L., Kobayashi T., Adachi S. (2012). Properties of Extract Obtained from Defatted Rice Bran by Extraction with Aqueous Ethanol under Subcritical Conditions. FSTR.

[15] Li Y., Qi Y., Li H., Chen Z., Xu B. (2022). Improving the Cold Water Swelling Properties of Oat Starch by Subcritical Ethanol-Water Treatment. Int. J. Biol. Macromol..

[16] De Souza A.R.C., Stefanov S., Bombardelli M.C.M., Corazza M.L., Stateva R.P. (2019). Assessment of Composition and Biological Activity of Arctium Lappa Leaves Extracts Obtained with Pressurized Liquid and Supercritical CO_2_ Extraction. J. Supercrit. Fluids.

[17] Tangkhavanich B., Kobayashi T., Adachi S. (2014). Effects of Repeated Treatment on the Properties of Rice Stem Extract Using Subcritical Water, Ethanol, and Their Mixture. J. Ind. Eng. Chem..

[18] FAO (2021). Citrus Fruit Statistical Compendium 2020.

[19] Kanaze F.I., Termentzi A., Gabrieli C., Niopas I., Georgarakis M., Kokkalou E. (2009). The Phytochemical Analysis and Antioxidant Activity Assessment of Orange Peel (*Citrus sinensis*) Cultivated in Greece—Crete Indicates a New Commercial Source of Hesperidin. Biomed. Chromatogr..

[20] Satari B., Karimi K. (2018). Citrus Processing Wastes: Environmental Impacts, Recent Advances, and Future Perspectives in Total Valorization. Resour. Conserv. Recycl..

[21] Ortiz-Sanchez M., Solarte-Toro J.C., Orrego-Alzate C.E., Acosta-Medina C.D., Cardona-Alzate C.A. (2021). Integral Use of Orange Peel Waste through the Biorefinery Concept: An Experimental, Technical, Energy, and Economic Assessment. Biomass Conv. Bioref..

[22] Laufenberg G., Kunz B., Nystroem M. (2003). Transformation of Vegetable Waste into Value Added Products. Bioresour. Technol..

[23] Sawalha S.M.S., Arráez-Román D., Segura-Carretero A., Fernández-Gutiérrez A. (2009). Quantification of Main Phenolic Compounds in Sweet and Bitter Orange Peel Using CE–MS/MS. Food Chem..

[24] Tripoli E., Guardia M.L., Giammanco S., Majo D.D., Giammanco M. (2007). Citrus Flavonoids: Molecular Structure, Biological Activity and Nutritional Properties: A Review. Food Chem..

[25] Yi L., Ma S., Ren D. (2017). Phytochemistry and Bioactivity of Citrus Flavonoids: A Focus on Antioxidant, Anti-Inflammatory, Anticancer and Cardiovascular Protection Activities. Phytochem. Rev..

[26] Apraj V., Pandita N. (2016). Evaluation of Skin Anti-Aging Potential of Citrus Reticulata Blanco Peel. Pharmacogn. Res..

[27] Stanisic D., Liu L.H.B., Dos Santos R.V., Costa A.F., Durán N., Tasic L. (2020). New Sustainable Process for Hesperidin Isolation and Anti-Ageing Effects of Hesperidin Nanocrystals. Molecules.

[28] Şahin D., Çağlar E.Ş., Boran T., Karadağ A.E., Özhan G., Üstündağ Okur N. (2023). Development, Characterization of Naringenin-Loaded Promising Microemulsion Formulations, and Demonstration of Anti-Aging Efficacy by in Vitro Enzyme Activity and Gene Expression. J. Drug Deliv. Sci. Technol..

[29] Nastić N., Vasić A., Šoronja Simović D., Vladić J., Jokić S., Aladić K., Vidović S. (2023). Underutilized Rosa Canina Herbal Dust as an Innovative Natural Functional and Health Promoting Ingredient: A Proposal of Two-Novel Approaches. Waste Biomass Valor..

[30] Živković J., Vladić J., Naffati A., Nastić N., Šavikin K., Tomić M., Vidović S. (2022). Comparative Chemical Profiling of Underexploited Arctostaphylos Uva-Ursi l. Herbal Dust Extracts Obtained by Conventional, Ultrasound-Assisted and Subcritical Water Extractions. Waste Biomass Valor..

[31] Sulejmanović M., Milić N., Mourtzinos I., Nastić N., Kyriakoudi A., Drljača J., Vidović S. (2024). Ultrasound-assisted and subcritical water extraction techniques for maximal recovery of phenolic compounds from raw ginger herbal dust toward in vitro biological activity investigation. Food Chem..

[32] Sánchez-Vallejo C., Ballesteros-Gómez A., Rubio S. (2022). Tailoring Composition and Nanostructures in Supramolecular Solvents: Impact on the Extraction Efficiency of Polyphenols from Vegetal Biomass. Sep. Purif. Technol..

[33] Popovic A., Drljaca J., Popovic M., Miljkovic D., Marinovic J., Ljubkovic M., Kladar N., Capo I. (2022). Mitochondrial Energy Metabolism in Baby Hamster Kidney (BHK-21/C13) Cells Treated with Karnozin Extra^®^. Int. J. Morphol..

[34] Crowley L.C., Christensen M.E., Waterhouse N.J. (2016). Measuring Survival of Adherent Cells with the Colony-Forming Assay. Cold Spring Harb. Protoc..

[35] Ma G., Zhang L., Sugiura M., Kato M. (2020). Citrus and Health. The Genus Citrus.

[36] Ignat I., Volf I., Popa V.I. (2011). A Critical Review of Methods for Characterisation of Polyphenolic Compounds in Fruits and Vegetables. Food Chem..

[37] Oboh G., Ademosun A.O. (2012). Characterization of the Antioxidant Properties of Phenolic Extracts from Some Citrus Peels. J. Food Sci. Technol..

[38] Guimarães R., Barros L., Barreira J.C.M., Sousa M.J., Carvalho A.M., Ferreira I.C.F.R. (2010). Targeting Excessive Free Radicals with Peels and Juices of Citrus Fruits: Grapefruit, Lemon, Lime and Orange. Food Chem. Toxicol..

[39] Plaza M., Turner C. (2015). Pressurized Hot Water Extraction of Bioactives. TrAC Trends Anal. Chem..

[40] Benito-Román Ó., Blanco B., Sanz M.T., Beltrán S. (2020). Subcritical Water Extraction of Phenolic Compounds from Onion Skin Wastes (*Allium Cepa* Cv. Horcal): Effect of Temperature and Solvent Properties. Antioxidants.

[41] Brezo-Borjan T., Švarc-Gajić J., Morais S., Delerue-Matos C., Rodrigues F., Lončarević I., Pajin B. (2023). Chemical and Biological Characterisation of Orange (*Citrus sinensis*) Peel Extracts Obtained by Subcritical Water. Processes.

[42] Razola-Díaz M.D.C., Guerra-Hernández E.J., Rodríguez-Pérez C., Gómez-Caravaca A.M., García-Villanova B., Verardo V. (2021). Optimization of Ultrasound-Assisted Extraction via Sonotrode of Phenolic Compounds from Orange by-Products. Foods.

[43] Krivošija S., Jerković I., Nastić N., Zloh M., Jokić S., Banožić M., Aladić K., Vidović S. (2023). Green Pathway for Utilisation of Orange Peel Dust and In Silico Evaluation of Pharmacological Potential. Microchem. J..

[44] M’hiri N., Irina I., Cédric P., Ghoul M., Boudhrioua N. (2017). Antioxidants of Maltease Orange Peel: Comparative Investigation of the Efficiency of Four Extraction Methods. J. Appl. Pharm. Sci..

[45] Londoño-Londoño J., Lima V.R.D., Lara O., Gil A., Pasa T.B.C., Arango G.J., Pineda J.R.R. (2010). Clean Recovery of Antioxidant Flavonoids from Citrus Peel: Optimizing an Aqueous Ultrasound-Assisted Extraction Method. Food Chem..

[46] Lachos-Perez D., Baseggio A.M., Torres-Mayanga P.C., Ávila P.F., Tompsett G.A., Marostica M., Goldbeck R., Timko M.T., Rostagno M., Martinez J. (2020). Sequential Subcritical Water Process Applied to Orange Peel for the Recovery Flavanones and Sugars. J. Supercrit. Fluids.

[47] Barrales F.M., Silveira P., Barbosa P.D.P.M., Ruviaro A.R., Paulino B.N., Pastore G.M., Macedo G.A., Martinez J. (2018). Recovery of Phenolic Compounds from Citrus By-Products Using Pressurized Liquids—An Application to Orange Peel. Food Bioprod. Process.

[48] Rafiq S., Kaul R., Sofi S.A., Bashir N., Nazir F., Ahmad Nayik G. (2018). Citrus Peel as a Source of Functional Ingredient: A Review. J. Saudi Soc. Agric. Sci..

[49] Hwang H.-J., Kim H.-J., Ko M.-J., Chung M.-S. (2021). Recovery of Hesperidin and Narirutin from Waste Citrus Unshiu Peel Using Subcritical Water Extraction Aided by Pulsed Electric Field Treatment. Food Sci. Biotechnol..

[50] Chen R., Qi Q.-L., Wang M.-T., Li Q.-Y. (2016). Therapeutic Potential of Naringin: An Overview. Phar Biol..

[51] Castro-Vázquez L., Lozano M.V., Rodríguez-Robledo V., González-Fuentes J., Marcos P., Villaseca N., Arroyo-Jiménez M.M., Santander-Ortega M.J. (2021). Pressurized Extraction as an Opportunity to Recover Antioxidants from Orange Peels: Heat Treatment and Nanoemulsion Design for Modulating Oxidative Stress. Molecules.

[52] Wittenauer J., Mäckle S., Sußmann D., Schweiggert-Weisz U., Carle R. (2015). Inhibitory Effects of Polyphenols from Grape Pomace Extract on Collagenase and Elastase Activity. Fitoterapia.

[53] Bielach-Bazyluk A., Zbroch E., Mysliwiec H., Rydzewska-Rosolowska A., Kakareko K., Flisiak I., Hryszko T. (2021). Sirtuin 1 and Skin: Implications in Intrinsic and Extrinsic Aging—A Systematic Review. Cells.

[54] Han K.-H., Choi H.-R., Won C.-H., Chung J.-H., Cho K.-H., Eun H.-C., Kim K.-H. (2005). Alteration of the TGF-β/SMAD Pathway in Intrinsically and UV-Induced Skin Aging. Mech. Ageing Dev..

[55] Gellibert F., Woolven J., Fouchet M.-H., Mathews N., Goodland H., Lovegrove V., Laroze A., Nguyen V.-L., Sautet S., Wang R. (2004). Identification of 1,5-Naphthyridine Derivatives as a Novel Series of Potent and Selective Tgf-β Type i Receptor Inhibitors. J. Med. Chem..

[56] Wang J., Fang X., Ge L., Cao F., Zhao L., Wang Z., Xiao W. (2018). Antitumor, Antioxidant and Anti-Inflammatory Activities of Kaempferol and Its Corresponding Glycosides and the Enzymatic Preparation of Kaempferol. PLoS ONE.

[57] Azeem M., Hanif M., Mahmood K., Ameer N., Chughtai F.R.S., Abid U. (2023). An Insight into Anticancer, Antioxidant, Antimicrobial, Antidiabetic and Anti-Inflammatory Effects of Quercetin: A Review. Polym. Bull..

[58] Liu L., Wu Y., Zhao Y., Lu C., Zhao R. (2023). Citric Acid Enhances the Activities of Astilbin on Psoriasis via Down-Regulation of *p* -Glycoprotein. Mol. Pharm..

[59] Yang Z., Teng Y., Wang H., Hou H. (2013). Enhancement of Skin Permeation of Bufalin by Limonene via Reservoir Type Transdermal Patch: Formulation Design and Biopharmaceutical Evaluation. Int. JPharm.

[60] Tisserand R., Young R. (2013). Essential Oil Safety: A Guide for Health Care Professionals.

[61] Hamuel J.D. (2015). The Occurrence, Properties and Significance of Citrinin Mycotoxin. J. Plant Pathol. Microbiol..

[62] Al Qaisi Y., Alfarrayeh I., Alsarayreh A., Khleifat K., Abu-Nwas N. (2024). Assessment of Antioxidant Potential, Cytotoxicity, and Anticancer Activity of Methanolic Extracts from Selected Wild Medicinal Plants. Phytomed. Plus.

[63] Barzegarparay F., Najafzadehvarzi H., Pourbagher R., Parsian H., Ghoreishi S.M., Mortazavi-Derazkola S. (2024). Green Synthesis of Novel Selenium Nanoparticles Using Crataegus Monogyna Extract (Senps@cm) and Investigation of Its Toxicity, Antioxidant Capacity, and Anticancer Activity against MCF-7 as a Breast Cancer Cell Line. Biomass Conv. Bioref..

[64] Jongrungraungchok S., Madaka F., Wunnakup T., Sudsai T., Pongphaew C., Songsak T., Pradubyat N. (2023). In Vitro Antioxidant, Anti-Inflammatory, and Anticancer Activities of Mixture Thai Medicinal Plants. BMC Complement. Med. Ther..

[65] Rani D.M., Wongso H., Purwoko R.Y., Winarto N.B., Shalas A.F., Triatmoko B., Pratama A.N.W., Keller P.A., Nugraha A.S. (2023). Anti-Cancer Bioprospecting on Medicinal Plants from Indonesia: A Review. Phytochemistry.

[66] Chen Z.-T., Chu H.-L., Chyau C.-C., Chu C.-C., Duh P.-D. (2012). Protective Effects of Sweet Orange (*Citrus sinensis*) Peel and Their Bioactive Compounds on Oxidative Stress. Food Chem..

[67] Banjerdpongchai R., Wudtiwai B., Khaw-on P., Rachakhom W., Duangnil N., Kongtawelert P. (2016). Hesperidin from Citrus Seed Induces Human Hepatocellular Carcinoma HepG2 Cell Apoptosis via Both Mitochondrial and Death Receptor Pathways. Tumor Biol..

[68] Bansal K., Bhati H., Vanshita, Bajpai M. (2024). New Insights into Therapeutic Applications and Nanoformulation Approaches of Hesperidin: An Updated Review. Pharmacol. Res.-Mod. Chin. Med..

[69] Zangade S.B., Dhulshette B.S., Patil P.B. (2024). Flavonoid-Metal Ion Complexes as Potent Anticancer Metallodrugs: A Comprehensive Review. Mini-Rev. Med. Chem..

[70] Hasnat H., Shompa S.A., Islam M.M., Alam S., Richi F.T., Emon N.U., Ashrafi S., Ahmed N.U., Chowdhury M.N.R., Fatema N. (2024). Flavonoids: A Treasure House of Prospective Pharmacological Potentials. Heliyon.

[71] Puteri A.R., Huda H.B. (2024). Flavonoids and Their Role As Anti-Inflammatory Agents. Biomed. J. Indones..

[72] Lobo C.L., Prabhu P.P., Dubey A., Shetty A., Mahadev M. (2024). Exploring the Potential of Hesperidin and Synergistic Formulations in Breast Cancer Management: A Comprehensive Review. Int. J. Pharm. Investig..

[73] Deng R., Liu Y., Wu X., Zhao N., Deng J., Pan T., Cao L., Zhan F., Qiao X. (2024). Probing the Interaction of Hesperidin Showing Antiproliferative Activity in Colorectal Cancer Cells and Human Hemoglobin. Int. J. Biol. Macromol..

[74] Bakhshan M.A., Sheikhzadeh S., Delirezh N. (2024). Hesperidin Nanoparticles for Prostate Cancer Therapy: Preparation, Characterization and Cytotoxic Activity. Biomed. Mater..

[75] Chang T.-M., Chi M.-C., Chiang Y.-C., Lin C.-M., Fang M.-L., Lee C.-W., Liu J.-F., Kou Y.R. (2024). Promotion of ROS-Mediated Apoptosis, G2/M Arrest, and Autophagy by Naringenin in Non-Small Cell Lung Cancer. Int. J. Biol. Sci..

[76] Elsori D., Pandey P., Ramniwas S., Kumar R., Lakhanpal S., Rab S.O., Siddiqui S., Singh A., Saeed M., Khan F. (2024). Naringenin as Potent Anticancer Phytocompound in Breast Carcinoma: From Mechanistic Approach to Nanoformulations Based Therapeutics. Front. Pharmacol..

[77] Ahmadi A., Shadboorestan A. (2016). Oxidative Stress and Cancer; the Role of Hesperidin, a Citrus Natural Bioflavonoid, as a Cancer Chemoprotective Agent. Nutr. Cancer.

[78] Bartoszewski R., Hering A., Marszałł M., Stefanowicz Hajduk J., Bartoszewska S., Kapoor N., Kochan K., Ochocka R. (2014). Mangiferin Has an Additive Effect on the Apoptotic Properties of Hesperidin in Cyclopia Sp. Tea Extracts. PLoS ONE.

[79] Park H.J., Kim M.-J., Ha E., Chung J.-H. (2008). Apoptotic Effect of Hesperidin through Caspase3 Activation in Human Colon Cancer Cells, SNU-C4. Phytomedicine.

[80] Abroon S., Nouri M., Mahdavi M. (2024). Hesperidin/Salinomycin Combination; a Natural Product for Deactivation of the Pi3k/Akt Signaling Pathway and Anti-Apoptotic Factors in Kg1a Cells. J. Fluoresc..

[81] Gavamukulya Y., Abou-Elella F., Wamunyokoli F., AEl-Shemy H. (2014). Phytochemical Screening, Anti-Oxidant Activity and in Vitro Anticancer Potential of Ethanolic and Water Leaves Extracts of Annona Muricata (Graviola). Asian Pac. J. Trop. Med..

