# Supplementary material for: Subcritical Water and Pressurised Ethanol Extractions for Maximum Recovery of Antioxidants from Orange Peel Herbal Dust with Evaluation of Its Pharmacological Potential Using In Silico and In Vitro Analysis
Source: Antioxidants (Basel). 2025 May 26;14(6):638. doi: 10.3390/antiox14060638 (PMC12189749; doi:10.3390/antiox14060638)
Supplement: Supplementary file 1 [file antioxidants-14-00638-s001.zip › antioxidants-3612375-supplementary.pdf]

Subcritical water and pressurised ethanol extractions for max-imum recovery of antioxidants from orange peel herbal dust with evaluation of its pharmacological potential using *in silico* and *in vitro* analysis

SUPPLEMENTARY INFORMATION

Slađana Krivošija[a], Ana Ballesteros-Gómez[b], Mire Zloh[c], Nataša Milić[d], Aleksandra Popović[e], Nataša Nastić[a], Senka Vidović[a*]

[a]*Department of Pharmaceutical Engineering, Faculty of Technology Novi Sad, University of Novi Sad, Boulevard cara Lazara 1, 21000 Novi Sad, Serbia*

[b]*Department of Analytical Chemistry, Institute of Chemistry for Energy and the Environment, University of Córdoba, 14071 Cordoba, Spain*

[c]*UCL School of Pharmacy, University College London, 29/39 Brunswick Square, London WC1N 1AX, UK*

[d]*Department of Pharmacy, Faculty of Medicine, University of Novi Sad, Hajduk Veljkova 3, 21000, Novi Sad, Serbia*

[e]*Department of Physiology, Faculty of Medicine, University of Novi Sad, Hajduk Veljkova 3, 21000, Novi Sad, Serbia*

[*]*Corresponding author, email:* senka.vidovic@uns.ac.rs

Table S1. Liquid chromatography-high resolution tandem mass spectrometry (LC-ESI-MS/MS) qualitative analysis of liquid orange peel dust (OPD) extracts.

Ions	Name	Molecular Formula	Extracts
[M-H]-	(9Z,12R)-12-Hydroxyoctadec-9-enoic acid	C18H34O3	PEE 1, PEE 2, PEE 3, PEE 4, PEE 5, PEE 6
[M-H]-	(Z)-prop-1-ene-1,2,3-tricarboxylic acid	C6H6O6	SWE 1, SWE 2, SWE 3, SWE 4, SWE 5, SWE 6
[M-H]-	1-Methoxy-3-carbaldehyde	C10H9NO2	PEE 1, PEE 2, PEE 3, PEE 4, PEE 5, PEE 6
[M-H]-	12-hydroxyoctadecanoic acid	C18H36O3	PEE 1, PEE 2, PEE 3, PEE 4, PEE 5, PEE 6
[M-H]-	13-HOTrE	C18H30O3	PEE 1, PEE 2, PEE 3, PEE 4, PEE 5, PEE 6
[M-H]-	2,4,6-Trimethylbenzoic acid	C10H12O2	PEE 1, PEE 2, PEE 3, PEE 4, PEE 5, PEE 6
[M-H]-	2,5-Dihydroxydioxane-2,5-dimethanol	C6H12O6	SWE 1, SWE 2, SWE 3, SWE 4, SWE 5, SWE 6
[M-H]-	2-Hydroxyphenylacetic acid	C8H8O3	PEE 1, PEE 2, PEE 3, PEE 4, PEE 5, PEE 6
[M-H]-	2-Methylglutaric acid	C6H10O4	PEE 1, PEE 2, PEE 3, PEE 4, PEE 5, PEE 6
[M-H]-	3,4-dihydroxybenzoic acid	C7H6O4	SWE 1, SWE 2, SWE 3, SWE 4, SWE 5, SWE 6, PEE 1, PEE 2, PEE 3, PEE 4, PEE 5, PEE 6
[M-H]-	3,4-Dimethoxycinnamic acid	C11H12O4	PEE 1, PEE 2, PEE 3, PEE 4, PEE 5, PEE 6
[M-H]-	3',5'-Dimethoxy-4'-hydroxyacetophenone	C10H12O4	SWE 1, SWE 2, SWE 3, SWE 4, SWE 5, SWE 6, PEE 1, PEE 2, PEE 3, PEE 4, PEE 5, PEE 6
[M-H]-	3,5-Dimethoxycinnamic acid	C11H12O4	PEE 1, PEE 2, PEE 3, PEE 4, PEE 5, PEE 6
[M-H]-	3,7-Dihydroxy-3',4'-dimethoxyflavone	C17H14O6	SWE 1, SWE 2, SWE 3, SWE 4, SWE 5, SWE 6, PEE 1, PEE 2, PEE 3, PEE 4, PEE 5, PEE 6
[M-H]-	3-Hydroxy-3',4',5'-trimethoxyflavone	C18H16O6	SWE 1, SWE 2, SWE 3, SWE 4, SWE 5, SWE 6, PEE 1, PEE 2, PEE 3, PEE 4, PEE 5, PEE 6

[M-H]-	3-Hydroxy-3-methylglutarate	C6H10O5	SWE 1, SWE 2, SWE 3, SWE 4, SWE 5, SWE 6, PEE 1, PEE 2, PEE 3, PEE 4, PEE 5, PEE 6
[M-H]-	3-hydroxybenzoic acid	C7H6O3	SWE 1, SWE 2, SWE 3, SWE 4, SWE 5, SWE 6, PEE 1, PEE 2, PEE 3, PEE 4, PEE 5, PEE 6
[M-H]-	3-hydroxy-C4-homoserine lactone	C8H13NO4	SWE 1, SWE 2, SWE 3, SWE 4, SWE 5, SWE 6, PEE 1, PEE 2, PEE 3, PEE 4, PEE 5, PEE 6
[M-H]-	3-Hydroxymandelic acid	C8H8O4	SWE 1, SWE 2, SWE 3, SWE 4, SWE 5, SWE 6, PEE 1, PEE 2, PEE 3, PEE 4, PEE 5, PEE 6
[M-H]-	3-Hydroxyphenylacetic acid	C8H8O3	PEE 1, PEE 2, PEE 3, PEE 4, PEE 5, PEE 6
[M-H]-	3-IAA	C10H9NO2	PEE 1, PEE 2, PEE 3, PEE 4, PEE 5, PEE 6
[M-H]-	4-Ethoxy-4-oxobut-2-enoic acid	C6H8O4	SWE 1, SWE 2, SWE 3, SWE 4, SWE 5, SWE 6, PEE 1, PEE 2, PEE 3, PEE 4, PEE 5, PEE 6
[M-H]-	4-Hydroxy-3,5-dimethoxy-cinnamic acid	C11H12O5	SWE 1, SWE 2, SWE 3, SWE 4, SWE 5, SWE 6
[M-H]-	4-Hydroxyacetophenone	C8H8O2	PEE 1, PEE 2, PEE 3, PEE 4, PEE 5, PEE 6
[M-H]-	4-hydroxybenzaldehyde	C7H6O2	PEE 1, PEE 2, PEE 3, PEE 4, PEE 5, PEE 6
[M-H]-	4-Hydroxyquinoline	C9H7NO	PEE 1, PEE 2, PEE 3, PEE 4, PEE 5, PEE 6
[M-H]-	4-Methoxycinnamic acid	C10H10O3	SWE 1, SWE 2, SWE 3, SWE 4, SWE 5, SWE 6, PEE 1, PEE 2, PEE 3, PEE 4, PEE 5, PEE 6
[M-H]-	4-methyl-2-oxopentanoic acid	C6H10O3	SWE 1, SWE 2, SWE 3, SWE 4, SWE 5, SWE 6
[M-H]-	4-Pyridoxate	C8H9NO4	SWE 1, SWE 2, SWE 3, SWE 4, SWE 5, SWE 6, PEE 1, PEE 2, PEE 3, PEE 4, PEE 5, PEE 6

[M-H]-	5,6-Dihydroxy-3',4'-dimethoxyflavanone	C17H16O6	SWE 1, SWE 2, SWE 3, SWE 4, SWE 5, SWE 6, PEE 1, PEE 2, PEE 3, PEE 4, PEE 5, PEE 6
[M-H]-	6,8-Dimethyl-4-hydroxycoumarin	C11H10O3	SWE 1, SWE 2, SWE 3, SWE 4, SWE 5, SWE 6, PEE 1, PEE 2, PEE 3, PEE 4, PEE 5, PEE 6
[M-H]-	6-Hydroxy-4-methylcoumarin, 6-Hydroxy-4-methyl-2-benzopyrone	C10H8O3	PEE 1, PEE 2, PEE 3, PEE 4, PEE 5, PEE 6
[M-H]-	6-Methoxy-7-hydroxycoumarin	C10H8O4	SWE 1, SWE 2, SWE 3, SWE 4, SWE 5, SWE 6, PEE 1, PEE 2, PEE 3, PEE 4, PEE 5, PEE 6
[M-H]-	6-Methoxyluteolin	C16H12O7	SWE 1, SWE 2, SWE 3, SWE 4, SWE 5, SWE 6, PEE 1, PEE 2, PEE 3, PEE 4, PEE 5, PEE 6
[M-H]-	7,8-Dihydroxycoumarin	C9H6O4	SWE 1, SWE 2, SWE 3, SWE 4, SWE 5, SWE 6, PEE 1, PEE 2, PEE 3, PEE 4, PEE 5, PEE 6
[M-H]-	7H-purin-6-amine	C5H5N5	SWE 1, SWE 2, SWE 3, SWE 4, SWE 5, SWE 6, PEE 1, PEE 2, PEE 3, PEE 4, PEE 5, PEE 6
[M-H]-	9Z,12Z-Linoleic acid (NMR)	C18H32O2	SWE 1, SWE 2, SWE 3, SWE 4, SWE 5, SWE 6, PEE 1, PEE 2, PEE 3, PEE 4, PEE 5, PEE 6
[M-H]-	Abscisic acid	C15H20O4	SWE 1, SWE 2, SWE 3, SWE 4, SWE 5, SWE 6, PEE 1, PEE 2, PEE 3, PEE 4, PEE 5, PEE 6
[M-H]-	AcetylPhenylalanine	C11H13NO3	SWE 1, SWE 2, SWE 3, SWE 4, SWE 5, SWE 6
[M-H]-	Apigenin	C15H10O5	PEE 1, PEE 2, PEE 3, PEE 4, PEE 5, PEE 6
[M-H]-	Aspirin	C9H8O4	SWE 1, SWE 2, SWE 3, SWE 4, SWE 5, SWE 6, PEE 1, PEE 2, PEE 3, PEE 4, PEE 5, PEE 6

[M-H]-	Azelaic acid (Not validated)	C9H16O4	SWE 1, SWE 2, SWE 3, SWE 4, SWE 5, SWE 6, PEE 1, PEE 2, PEE 3, PEE 4, PEE 5, PEE 6
[M-H]-	C12-AE1S (TENTATIVE)	C14H30O5S	SWE 1, SWE 2, SWE 3, SWE 4, SWE 5, SWE 6, PEE 1, PEE 2, PEE 3, PEE 4, PEE 5, PEE 6
[M-H]-	Caffeoyl quinic acid (isomer of 831, 833, 834)	C16H18O9	PEE 1, PEE 2, PEE 3, PEE 4, PEE 5, PEE 6
[M-H]-	Caffeyl alcohol	C9H10O3	SWE 1, SWE 2, SWE 3, SWE 4, SWE 5, SWE 6, PEE 1, PEE 2, PEE 3, PEE 4, PEE 5, PEE 6
[M-H]-	Catechin(+)	C15H14O6	PEE 1, PEE 2, PEE 3, PEE 4, PEE 5, PEE 6
[M-H]-	Catechol	C6H6O2	SWE 1, SWE 2, SWE 3, SWE 4, SWE 5, SWE 6, PEE 1, PEE 2, PEE 3, PEE 4, PEE 5, PEE 6
[M-H]-	Chalcone base + 2O, 1MeO, 1Prenyl or Licochalcone A (not validated)	C21H22O4	PEE 1, PEE 2, PEE 3, PEE 4, PEE 5, PEE 6
[M-H]-	Citramalic acid	C5H8O5	SWE 1, SWE 2, SWE 3, SWE 4, SWE 5, SWE 6, PEE 1, PEE 2, PEE 3, PEE 4, PEE 5, PEE 6
[M-H]-	citric acid	C6H8O7	SWE 1, SWE 2, SWE 3, SWE 4, SWE 5, SWE 6
[M-H]-	Citric acid (Not validated, isomer of 227)	C6H8O7	SWE 1, SWE 2, SWE 3, SWE 4, SWE 5, SWE 6, PEE 1, PEE 2, PEE 3, PEE 4, PEE 5, PEE 6
[M-H]-	Citrinin	C13H14O5	PEE 1, PEE 2, PEE 3, PEE 4, PEE 5, PEE 6
[M-H]-	Coniferyl aldehyde	C10H10O3	SWE 1, SWE 2, SWE 3, SWE 4, SWE 5, SWE 6, PEE 1, PEE 2, PEE 3, PEE 4, PEE 5, PEE 6
[M-H]-	Coumaric acid (isomer of 131)	C9H8O3	SWE 1, SWE 2, SWE 3, SWE 4, SWE 5, SWE 6, PEE 1, PEE 2, PEE 3, PEE 4, PEE 5, PEE 6

[M-H]-	Coumaroyl Hexoside (isomer of 690, 691)	C15H18O8	SWE 1, SWE 2, SWE 3, SWE 4, SWE 5, SWE 6, PEE 1, PEE 2, PEE 3, PEE 4, PEE 5, PEE 6
[M-H]-	Ctrinin hydrate	C13H16O6	PEE 1, PEE 2, PEE 3, PEE 4, PEE 5, PEE 6
[M-H]-	D(-)-Gulono-gamma-lactone	C6H10O6	SWE 1, SWE 2, SWE 3, SWE 4, SWE 5, SWE 6, PEE 1, PEE 2, PEE 3, PEE 4, PEE 5, PEE 6
[M-H]-	D-(-)-quinic acid	C7H12O6	SWE 1, SWE 2, SWE 3, SWE 4, SWE 5, SWE 6, PEE 1, PEE 2, PEE 3, PEE 4, PEE 5, PEE 6
[M-H]-	D(+)-Galacturonic acid	C6H10O7	SWE 1, SWE 2, SWE 3, SWE 4, SWE 5, SWE 6, PEE 1, PEE 2, PEE 3, PEE 4, PEE 5, PEE 6
[M-H]-, [M-H-H2O]-	D-(+)-Malic acid	C4H6O5	SWE 1, SWE 2, SWE 3, SWE 4, SWE 5, SWE 6, PEE 1, PEE 2, PEE 3, PEE 4, PEE 5, PEE 6
[M-H]-	Datiscetin-3-O-rutinoside	C27H30O15	SWE 1, SWE 2, SWE 3, SWE 4, SWE 5, SWE 6, PEE 1, PEE 2, PEE 3, PEE 4, PEE 5
[M-H]-	Decanedioic acid	C10H18O4	SWE 1, SWE 2, SWE 3, SWE 4, SWE 5, SWE 6
[M-H]-	D-Galactose	C6H12O6	SWE 1, SWE 2, SWE 3, SWE 4, SWE 5, SWE 6, PEE 1, PEE 2, PEE 3, PEE 4, PEE 5, PEE 6
[M-H]-	D-Gluconic acid	C6H12O7	SWE 1, SWE 2, SWE 3, SWE 4, SWE 5, SWE 6
[M-H]-	Diosmin	C28H32O15	SWE 1, SWE 2, SWE 3, SWE 4, SWE 5, PEE 1, PEE 2, PEE 3, PEE 4, PEE 5, PEE 6
[M-H]-	DL-3,4-Dihydroxymandelic acid	C8H8O5	SWE 1, SWE 2, SWE 3, SWE 4, SWE 5, SWE 6, PEE 1, PEE 2, PEE 3, PEE 4, PEE 5, PEE 6
[M-H]-	DL-4-Hydroxy-3-methoxymandelic acid	C9H10O5	PEE 1, PEE 2, PEE 3, PEE 4, PEE 5, PEE 6

[M-H]-	DL-p-Hydroxyphenyllactic acid	C9H10O4	SWE 1, SWE 2, SWE 3, SWE 4, SWE 5, SWE 6, PEE 1, PEE 2, PEE 3, PEE 4, PEE 5, PEE 6
[M-H]-	Dodecanedioic acid	C12H22O4	SWE 1, SWE 2, SWE 3, SWE 4, SWE 5, SWE 6, PEE 1, PEE 2, PEE 3, PEE 4, PEE 5, PEE 6
[M-H]-	D-Ribose	C5H10O5	PEE 1, PEE 2, PEE 3, PEE 4, PEE 5, PEE 6
[M-H]-	D-Tartaric acid	C4H6O6	SWE 1, SWE 2, SWE 3, SWE 4, SWE 5, SWE 6, PEE 1, PEE 2, PEE 3, PEE 4, PEE 5, PEE 6
[M-H]-	Epigallocatechin	C15H14O7	PEE 1, PEE 2, PEE 3, PEE 4, PEE 5, PEE 6
[M-H]-	esculetin	C9H6O4	SWE 1, SWE 2, SWE 3, SWE 4, SWE 5, SWE 6
[M-H]-	Ethyl vanillate	C10H12O4	SWE 1, SWE 2, SWE 3, SWE 4, SWE 5, SWE 6
[M-H]-	FA 13:3+1O	C13H20O3	SWE 1, SWE 2, SWE 3, SWE 4, SWE 5, SWE 6, PEE 1, PEE 2, PEE 3, PEE 4, PEE 5, PEE 6
[M-H]-	FA 18:1+2O	C18H34O4	PEE 1, PEE 2, PEE 3, PEE 4, PEE 5, PEE 6
[M-H]-	FA 18:2+1O	C18H32O3	PEE 1, PEE 2, PEE 3, PEE 4, PEE 5, PEE 6
[M-H]-	FA 18:2+2O	C18H32O4	SWE 1, SWE 2, SWE 3, SWE 4, SWE 5, SWE 6, PEE 1, PEE 2, PEE 3, PEE 4, PEE 5, PEE 6
[M-H]-	FA 18:2+3O	C18H32O5	SWE 1, SWE 2, SWE 3, SWE 4, SWE 5, SWE 6, PEE 1, PEE 2, PEE 3, PEE 4, PEE 5, PEE 6
[M-H]-	FA 18:3+1O	C18H30O3	PEE 1, PEE 2, PEE 3, PEE 4, PEE 5, PEE 6
[M-H]-	FA 18:3+2O	C18H30O4	PEE 1, PEE 2, PEE 3, PEE 4, PEE 5, PEE 6
[M-H]-	FA 18:3+3O	C18H30O5	SWE 1, SWE 2, SWE 3, SWE 4, SWE 5, SWE 6
[M-H]-	FA 18:4+2O	C18H30O4	PEE 1, PEE 2, PEE 3, PEE 4, PEE 5, PEE 6

[M-H]-	FA 9:0+1O	C9H18O3	PEE 1, PEE 2, PEE 3, PEE 4, PEE 5, PEE 6
[M-H]-	Feruloyl Hexoside (isomer of 847)	C16H20O9	SWE 1, SWE 2, SWE 3, SWE 4, SWE 5, SWE 6, PEE 1, PEE 2, PEE 3, PEE 4, PEE 5, PEE 6
[M-H]-	Feruloyl quinic acid (isomer of 887, 888)	C17H20O9	SWE 1, SWE 2, SWE 3, SWE 4, SWE 5, SWE 6
[M-H]-	Flavone base + 3O, 2MeO	C17H14O7	SWE 1, SWE 2, SWE 3, SWE 4, SWE 5, SWE 6, PEE 1, PEE 2, PEE 3, PEE 4, PEE 5, PEE 6
[M-H]-	Flavone base + 3O, C-Hex, C-Pen	C26H28O14	PEE 1, PEE 2, PEE 3, PEE 4, PEE 5, PEE 6
[M-H]-	Flavone base + 4O, C-(dehydro-dHex)-dHex	C27H28O14	PEE 1, PEE 2, PEE 3, PEE 4, PEE 5, PEE 6
[M-H]-	Flavonol base + 4O, 1MeO	C16H12O8	PEE 1, PEE 2, PEE 3, PEE 4, PEE 5, PEE 6
[M-H]-	Fraxetin	C10H8O5	SWE 1, SWE 2, SWE 3, SWE 4, SWE 5, SWE 6, PEE 1, PEE 2, PEE 3, PEE 4, PEE 5, PEE 6
[M-H]-	Fustin	C15H12O6	PEE 1, PEE 2, PEE 3, PEE 4, PEE 5, PEE 6
[M-H]-	Glutaric acid (Not validated)	C5H8O4	SWE 1, SWE 2, SWE 3, SWE 4, SWE 5, SWE 6, PEE 1, PEE 2, PEE 3, PEE 4, PEE 5, PEE 6
[M-H]-	Hesperetin	C16H14O6	SWE 1, SWE 2, SWE 3, SWE 4, SWE 5, SWE 6, PEE 1, PEE 2, PEE 3, PEE 4, PEE 5, PEE 6
[M-H]-, [M+Cl]-	Hesperidin	C28H34O15	SWE 1, SWE 2, SWE 3, SWE 4, SWE 5, SWE 6, PEE 1, PEE 2, PEE 3, PEE 4, PEE 5, PEE 6
[M-H]-	Hexose + C13H17O3	C19H28O9	SWE 1, SWE 2, SWE 3, SWE 4, SWE 5, SWE 6
[M-H]-	Homogentisic acid	C8H8O4	SWE 1, SWE 2, SWE 3, SWE 4, SWE 5, SWE 6, PEE 1, PEE 2, PEE 3, PEE 4, PEE 5, PEE 6

[M-H]-	Homovanillic acid	C9H10O4	SWE 1, SWE 2, SWE 3, SWE 4, SWE 5, SWE 6, PEE 1, PEE 2, PEE 3, PEE 4, PEE 5, PEE 6
[M-H]-	Hydrocerol A	C6H8O7	PEE 1, PEE 2, PEE 3, PEE 4, PEE 5, PEE 6
[M-H]-	Hydroxyferulic acid	C10H10O5	SWE 1, SWE 2, SWE 3, SWE 4, SWE 5, SWE 6, PEE 1, PEE 2, PEE 3, PEE 4, PEE 5, PEE 6
[M-H]-	Hydroxysebacic acid	C10H18O5	PEE 1, PEE 2, PEE 3, PEE 4, PEE 5, PEE 6
[M-H]-	Hydroxysuberic acid	C8H14O5	PEE 1, PEE 2, PEE 3, PEE 4, PEE 5, PEE 6
[M-H]-	Indole-3-acetic acid	C10H9NO2	PEE 1, PEE 2, PEE 3, PEE 4, PEE 5, PEE 6
[M-H]-	Indole-3-carboxyaldehyde	C9H7NO	SWE 1, SWE 2, SWE 3, SWE 4, SWE 5, SWE 6
[M-H]-, [M-H-H2O]-	Isocitrate	C6H8O7	PEE 1, PEE 2, PEE 3, PEE 4, PEE 5, PEE 6
[M-H]-	isofraxidin	C11H10O5	PEE 1, PEE 2, PEE 3, PEE 4, PEE 5, PEE 6
[M-H]-	Isosakuranetin	C16H14O5	SWE 1, SWE 2, SWE 3, SWE 4, SWE 5, SWE 6, PEE 1, PEE 2, PEE 3, PEE 4, PEE 5, PEE 6
[M-H]-, [M+Cl]-	isosakuranetin-7-O-rutinoside	C28H34O14	SWE 1, SWE 2, SWE 3, SWE 4, SWE 5
[M-H]-	Isovitexin(4)	C21H20O10	PEE 1, PEE 2, PEE 3, PEE 4, PEE 5, PEE 6
[M-H]-	Kaempferol-4'-methyl ether	C16H12O6	SWE 1, SWE 2, SWE 3, SWE 4, SWE 5, SWE 6, PEE 1, PEE 2, PEE 3, PEE 4, PEE 5, PEE 6
[M-H]-	Kaempferol-7-O-neohesperidoside	C27H30O15	SWE 1, SWE 2, SWE 3, SWE 4, SWE 5
[M-H]-	Limocitrin	C17H14O8	SWE 1, SWE 2, SWE 3, SWE 4, SWE 5, SWE 6, PEE 1, PEE 2, PEE 3, PEE 4, PEE 5, PEE 6
[M-H]-	Luteolin	C15H10O6	SWE 3, SWE 4, SWE 5, SWE 6, PEE 1, PEE 2, PEE 3, PEE 4, PEE 5, PEE 6

[M-H]-	luteolin-6-C-glucoside	C21H20O11	SWE 1, SWE 2, SWE 3, SWE 4, SWE 5, SWE 6
[M-H]-	Mesaconic acid	C5H6O4	SWE 1, SWE 2, SWE 3, SWE 4, SWE 5, SWE 6, PEE 1, PEE 2, PEE 3, PEE 4, PEE 5, PEE 6
[M-H]-	Methyl jasmonate	C13H20O3	SWE 1, SWE 2, SWE 3, SWE 4, SWE 5, SWE 6
[M-H]-	Methyl salicylate	C8H8O3	SWE 1, SWE 2, SWE 3, SWE 4, SWE 5, SWE 6
[M-H]-	Naringenin	C15H12O5	SWE 1, SWE 2, SWE 3, SWE 4, SWE 5, SWE 6, PEE 1, PEE 2, PEE 3, PEE 4, PEE 5, PEE 6
[M-H]-	Naringenin-7-O-glucoside	C21H22O10	SWE 1, SWE 2, SWE 3, SWE 4, SWE 5, SWE 6, PEE 1, PEE 2, PEE 3, PEE 4, PEE 5, PEE 6
[M-H]-	Naringenin-7-O-rutinoside	C27H32O14	PEE 1, PEE 2, PEE 3, PEE 4, PEE 5, PEE 6
[M-H]-	Nevadensin	C18H16O7	PEE 1, PEE 2, PEE 3, PEE 4, PEE 5, PEE 6
[M-H]-	Palmitic acid (NMR)	C16H32O2	SWE 1, SWE 2, SWE 3, SWE 4, SWE 5, SWE 6, PEE 1, PEE 2, PEE 3, PEE 4, PEE 5, PEE 6
[M-H]-	p-Coumaraldehyde	C9H8O2	SWE 1, SWE 2, SWE 3, SWE 4, SWE 5, SWE 6, PEE 1, PEE 2, PEE 3, PEE 4, PEE 5, PEE 6
[M-H]-	p-Coumaryl alcohol	C9H10O2	PEE 1, PEE 2, PEE 3, PEE 4, PEE 5, PEE 6
[M-H]-	Peonidin-3-O-beta-galactoside	C22H22O11	PEE 1, PEE 2, PEE 3, PEE 4, PEE 5, PEE 6
[M-H]-	Phenylacetic acid + 2O, O-Hex	C14H18O9	PEE 1, PEE 2, PEE 3, PEE 4, PEE 5, PEE 6
[M-H]-	Propyl gallate	C10H12O5	PEE 1, PEE 2, PEE 3, PEE 4, PEE 5, PEE 6
[M-H]-	Protocatechuic aldehyde	C7H6O3	SWE 1, SWE 2, SWE 3, SWE 4, SWE 5, SWE 6, PEE 1, PEE 2, PEE 3, PEE 4, PEE 5, PEE 6
[M-H]-	Quercetin	C15H10O7	SWE 2, SWE 3, SWE 4, SWE 5, SWE 6, PEE 1, PEE 2, PEE 3, PEE 4, PEE 5, PEE 6

[M-H]-	Robinetin trimethyl ether	C18H16O7	SWE 1, SWE 2, SWE 3, SWE 4, SWE 5, SWE 6, PEE 1, PEE 2, PEE 3, PEE 4, PEE 5, PEE 6
[M-H]-	Rutin	C27H30O16	SWE 1, SWE 2, SWE 3, SWE 4, SWE 5, SWE 6, PEE 1, PEE 2, PEE 3, PEE 4, PEE 5
[M-H]-	Saccharolactic acid	C6H10O8	SWE 1, SWE 2, SWE 3, SWE 4, SWE 5, SWE 6, PEE 1, PEE 2, PEE 3, PEE 4, PEE 5, PEE 6
[M-H]-	Salicylamide	C7H7NO2	SWE 1, SWE 2, SWE 3, SWE 4, SWE 5, SWE 6, PEE 1, PEE 2, PEE 3, PEE 4, PEE 5, PEE 6
[M-H]-	Salicylic acid	C7H6O3	SWE 1, SWE 2, SWE 3, SWE 4, SWE 5, SWE 6, PEE 1, PEE 2, PEE 3, PEE 4, PEE 5, PEE 6
[M-H]-	Scaposin	C19H18O9	SWE 2, SWE 3, SWE 4, SWE 5, SWE 6
[M-H]-	Scoparin	C22H22O11	SWE 1, SWE 2, SWE 3, SWE 4, SWE 5, SWE 6
[M-H]-	Shikimic acid	C7H10O5	SWE 1, SWE 2, SWE 3, SWE 4, SWE 5, SWE 6, PEE 1, PEE 2, PEE 3, PEE 4, PEE 5, PEE 6
[M-H]-	Sinapic acid	C11H12O5	SWE 1, SWE 2, SWE 3, SWE 4, SWE 5, SWE 6
[M-H]-	Sinapoyl aldehyde	C11H12O4	SWE 1, SWE 2, SWE 3, SWE 4, SWE 5, SWE 6, PEE 1, PEE 2, PEE 3, PEE 4, PEE 5, PEE 6
[M-H]-	Sinapoylhexoside (isomer of 953)	C17H22O10	SWE 1, SWE 2, SWE 3, SWE 4, SWE 5, SWE 6, PEE 1, PEE 2, PEE 3, PEE 4, PEE 5, PEE 6
[M-H]-	Suberic acid	C8H14O4	PEE 1, PEE 2, PEE 3, PEE 4, PEE 5, PEE 6
[M-H]-	Succinic acid	C4H6O4	PEE 1, PEE 2, PEE 3, PEE 4, PEE 5, PEE 6
[M-H]-	Sucrose	C12H22O11	PEE 1, PEE 2, PEE 3, PEE 4, PEE 5, PEE 6

[M-H]-	Syringetin-3-O-galactoside	C23H24O13	SWE 1, SWE 2, SWE 3, SWE 4, SWE 5, PEE 1, PEE 2, PEE 3, PEE 4, PEE 5, PEE 6
[M-H]-	Syringetin-3-O-glucoside	C23H24O13	SWE 1, SWE 2, SWE 3, SWE 4, SWE 5, SWE 6
[M-H]-	Syringic aldehyde	C9H10O4	SWE 1, SWE 2, SWE 3, SWE 4, SWE 5, PEE 1, PEE 2, PEE 3, PEE 4, PEE 5, PEE 6
[M-H]-	trans-4-Coumaric acid	C9H8O3	SWE 1, SWE 2, SWE 3, SWE 4, SWE 5, SWE 6, PEE 1, PEE 2, PEE 3, PEE 4, PEE 5, PEE 6
[M-H]-	trans-Caffeic acid	C9H8O4	SWE 1, SWE 2, SWE 3, SWE 4, SWE 5, SWE 6, PEE 1, PEE 2, PEE 3, PEE 4, PEE 5, PEE 6
[M-H]-	Trans-Vaccenic acid	C18H34O2	PEE 1, PEE 2, PEE 3, PEE 4, PEE 5, PEE 6
[M-H]-	Umbelliferone	C9H6O3	SWE 1, SWE 2, SWE 3, SWE 4, SWE 5, SWE 6, PEE 1, PEE 2, PEE 3, PEE 4, PEE 5, PEE 6
[M-H]-	Undecanedioic acid	C11H20O4	PEE 1, PEE 2, PEE 3, PEE 4, PEE 5, PEE 6
[M-H]-	Vanillic acid	C8H8O4	SWE 1, SWE 2, SWE 3, SWE 4, SWE 5, SWE 6, PEE 1, PEE 2, PEE 3, PEE 4, PEE 5, PEE 6
[M-H]-	Vanillin	C8H8O3	SWE 1, SWE 2, SWE 3, SWE 4, SWE 5, SWE 6, PEE 1, PEE 2, PEE 3, PEE 4, PEE 5, PEE 6

* SWE – Subcritical water extraction; PEE – Pressurised ethanol extraction.

Identification criteria: mass accuracy below or equal to 10 ppm, isotopic pattern fit below or equal to 200 mSigma and MS fragmentation score above 900.

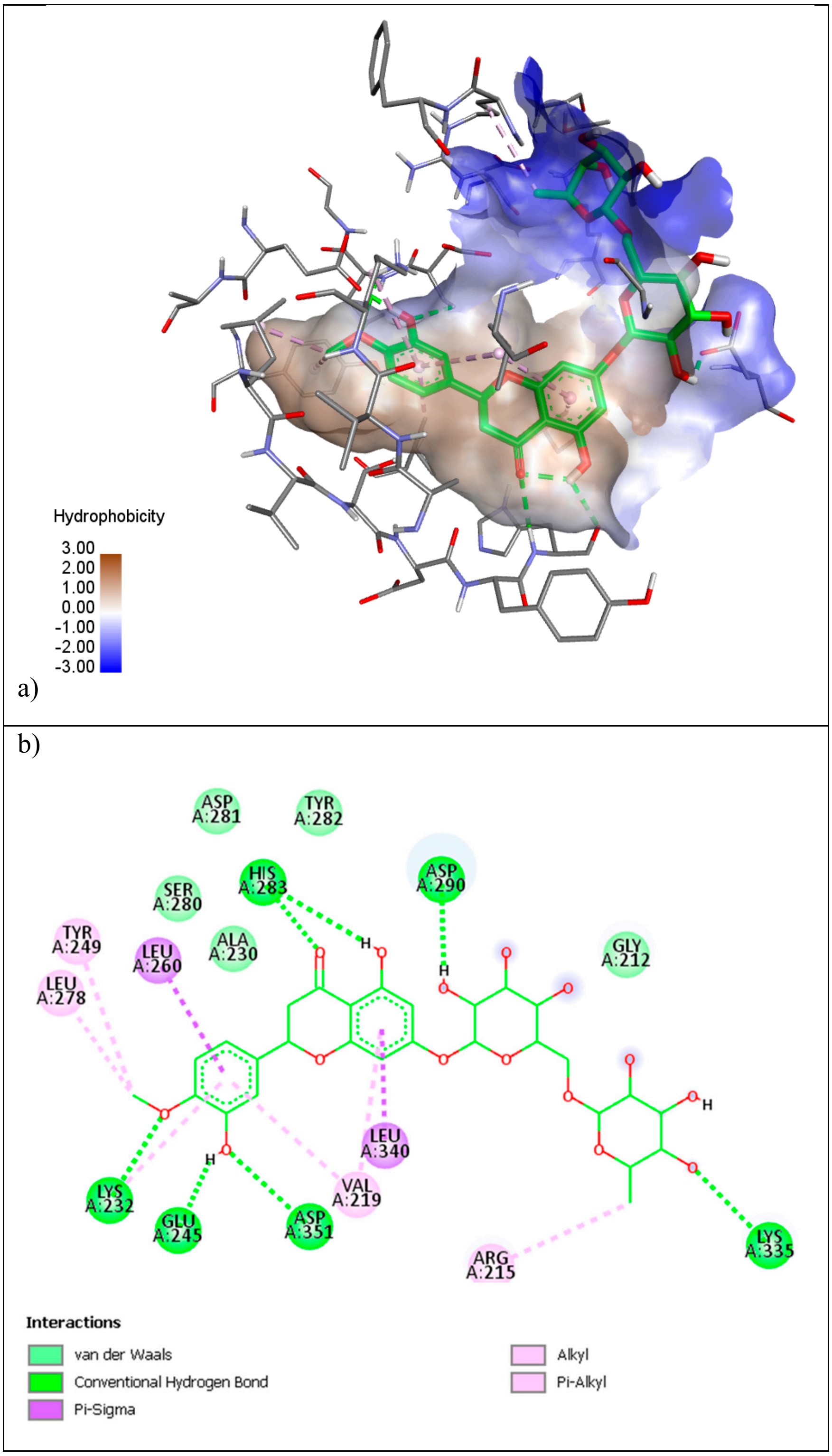

Figure S1. A representative docked pose of hesperidin into the binding site of growth factor beta receptor (surface coloured according to hydrophobicity) shown in as a) three dimensional representation of a ligand (thick green sticks) and b) two dimensional protein ligand plot.

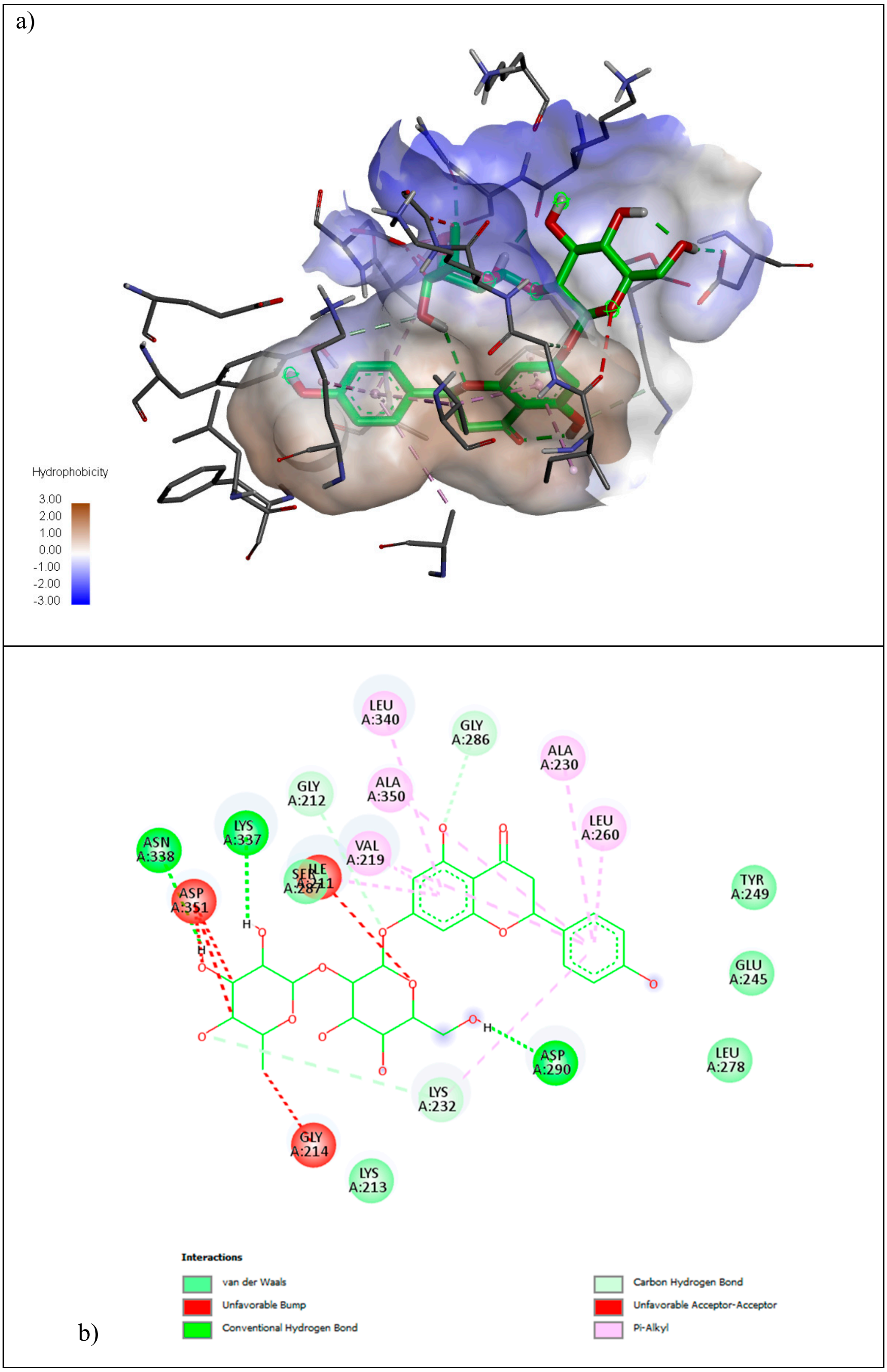

Figure S2. A representative docked pose of naringin into the binding site of growth factor beta receptor (surface coloured according to hydrophobicity) shown in as a) three dimensional representation of a ligand (thick green sticks) and b) two dimensional protein ligand plot.
